# Constructing gene co-expression networks and predicting functions of unknown genes by random matrix theory

**DOI:** 10.1186/1471-2105-8-299

**Published:** 2007-08-14

**Authors:** Feng Luo, Yunfeng Yang, Jianxin Zhong, Haichun Gao, Latifur Khan, Dorothea K Thompson, Jizhong Zhou

**Affiliations:** 1 Environmental Sciences Division, Oak Ridge National Laboratory, Oak Ridge, Tennessee 37831, USA; 2 Computer Science & Mathematics Division, Oak Ridge National Laboratory, Oak Ridge, Tennessee 37831, USA; 3 Department of Physics, Xiangtan University, Hunan 411105, PR China; 4 Department of Computer Science, University of Texas at Dallas, Richardson, TX 75083, USA; 5 School of Computing, Clemson University, Clemson, SC, 29634, USA; 6 Department of Biological Sciences, Purdue University, West Lafayette, IN, 47907, USA; 7 Insitute for Environmental Genomics, and Department of Botany and Microbiology, University of Oklahoma, Norman, OK, 73019, USA

## Abstract

**Background:**

Large-scale sequencing of entire genomes has ushered in a new age in biology. One of the next grand challenges is to dissect the cellular networks consisting of many individual functional modules. Defining co-expression networks without ambiguity based on genome-wide microarray data is difficult and current methods are not robust and consistent with different data sets. This is particularly problematic for little understood organisms since not much existing biological knowledge can be exploited for determining the threshold to differentiate true correlation from random noise. Random matrix theory (RMT), which has been widely and successfully used in physics, is a powerful approach to distinguish system-specific, non-random properties embedded in complex systems from random noise. Here, we have hypothesized that the universal predictions of RMT are also applicable to biological systems and the correlation threshold can be determined by characterizing the correlation matrix of microarray profiles using random matrix theory.

**Results:**

Application of random matrix theory to microarray data of *S. oneidensis*, *E. coli*, yeast, *A. thaliana*, *Drosophila*, mouse and human indicates that there is a sharp transition of nearest neighbour spacing distribution (NNSD) of correlation matrix after gradually removing certain elements insider the matrix. Testing on an *in silico *modular model has demonstrated that this transition can be used to determine the correlation threshold for revealing modular co-expression networks. The co-expression network derived from yeast cell cycling microarray data is supported by gene annotation. The topological properties of the resulting co-expression network agree well with the general properties of biological networks. Computational evaluations have showed that RMT approach is sensitive and robust. Furthermore, evaluation on sampled expression data of an *in silico *modular gene system has showed that under-sampled expressions do not affect the recovery of gene co-expression network. Moreover, the cellular roles of 215 functionally unknown genes from yeast, *E. coli *and *S. oneidensis *are predicted by the gene co-expression networks using guilt-by-association principle, many of which are supported by existing information or our experimental verification, further demonstrating the reliability of this approach for gene function prediction.

**Conclusion:**

Our rigorous analysis of gene expression microarray profiles using RMT has showed that the transition of NNSD of correlation matrix of microarray profile provides a profound theoretical criterion to determine the correlation threshold for identifying gene co-expression networks.

## Background

The cellular system, similar to engineering systems, is modular [1]. Hartwell et al. defined a module in biological system as "a discrete unit whose function is separable from those of other modules" and suggested that the functional modules are a "critical level of biological organization" [1]. One of important characteristics of modular system is collectivity. Namely, the similarities of behaviour or properties between elements in the same module are significantly higher than similarities between elements from different modules. Moreover, the cell is a complex system with many functionally diverse elements, including proteins, DNA, RNA and small molecules. Cellular functionalities involve groups of molecules interacting to each other. Modelling cellular systems as networks with connected elements allows us to understand the properties of cellular systems [2,3]. Thus, a module in a biological network can be defined as a sub-network that structurally has more insider links than outsider links and functionally is enriched with genes (proteins) in the same functional module.

The microarray technology, which enables massive parallel measurement of expressions of thousands of genes simultaneously, has opened up great opportunities for the systems-level understanding and elucidating of gene networks [4-6]. Various methods have been developed for inferring gene networks, such as differential equation-based network methods [7-10], Bayesian network methods [11,12] and relevance/co-expression network methods [13-15]. Nevertheless, the inference of genome-wide gene networks currently is still constrained by the dimensionality problem, namely, number of genes is far greater than the number of experiments in microarray data.

Because of its computational simplicity and the nature of microarray data (typically noisy, highly dimensional and significantly under-sampled) [16], co-expression network methods are most commonly used for identifying cellular networks [14,15,17-19]. As the expressions of genes in the same function modules generally are highly correlated, gene functional modules can be revealed from gene co-expression network as network modules. The co-expression network methods first construct a correlation matrix of gene expressions, in which the Pearson correlation and the mutual information are often used. Then, the co-expression network methods assign a link to a pair of genes when the correlation between their expressions exceeds a threshold [14,15,17-19]. Consequently, the network structure and topology, e.g. the number, size, content and connections of modules, are subjective, depending on the thresholds chosen. Thus, it is critical to appropriately define the threshold of correlation. Currently, thresholds are usually determined by either known biological information [17-19], or by statistical comparison to randomized expression data [20,21]. New approaches are urgently needed to determine gene networks in an automatic and objective fashion [3]. To tackle this, we developed a novel random matrix theory (RMT)-based approach to determine the threshold in this report.

Initially proposed by Wigner and Dyson in the 1960s for studying the spectrum of complex nuclei [22], RMT is a powerful approach for identifying and modelling phase transitions associated with disorder and noise in statistical physics and materials science. It has been successfully used for studying the behaviour of complex systems, such as spectra of large atoms [23], metal insulator transitions in disorder systems [24,25], spectra of quasiperiodic systems [23,26,27], chaotic systems [28], brain response [29] and the stock market [30]. However, its suitability for biological systems remains largely unexplored.

RMT makes two universal predictions for real symmetric matrices: the nearest neighbour spacing distribution (NNSD) of eigenvalues (i.e., the distribution of the difference of two nearest neighbour eigenvalues) follows Gaussian orthogonal ensemble (GOE) statistics if there exists correlation between nearest-neighbour eigenvalues, while it follows Poisson statistics if there is no correlation [23]. Deviations from GOE universal prediction can be used to distinguish system-specific, non-random properties of complex systems from random noise [30]. It has been well recognized that only a portion of genes change their expressions under different experimental conditions. Thus, the correlation matrix of gene expressions is the combination of the high correlation part Mc, which signifies the correlation of gene expressions specified to changes in biological systems, and the weak correlation part Mr or so-called noise, which signifies random relations between gene expressions: M = Mc + Mr. The modularity of the cellular systems indicates that Mc is non-random and will emerge collectivity property. Based on RMT, we hypothesized that the two universal predictions are applicable to biological systems. The NNSD of M will follow GOE and the NNSD of Mc will follow Poisson distribution. The transition of NNSD between GOE and Poisson distributions can serve as a reference point to distinguish system-specific, non-random relationship embedded in correlation matrix of gene microarray data from random noise. This reference point is mathematically defined and can be used as a threshold to identify gene co-expression networks in an automatic and objective fashion.

In this report, we describe the development and application of an RMT-based approach to determine the correlation threshold for identifying co-expression networks based on the microarray data from such simple-to-complex organisms as *S. oneidensis*, *E. coli*, yeast, *A. thaliana*, *Drosophila*, mouse and human. Moreover, the resulting co-expression networks are useful for predicting the function of unknown genes, which is supported by existing information and our experimental verification.

## Results

### *In silico *evaluation of RMT approach to determine correlation threshold

**Figure 1:**
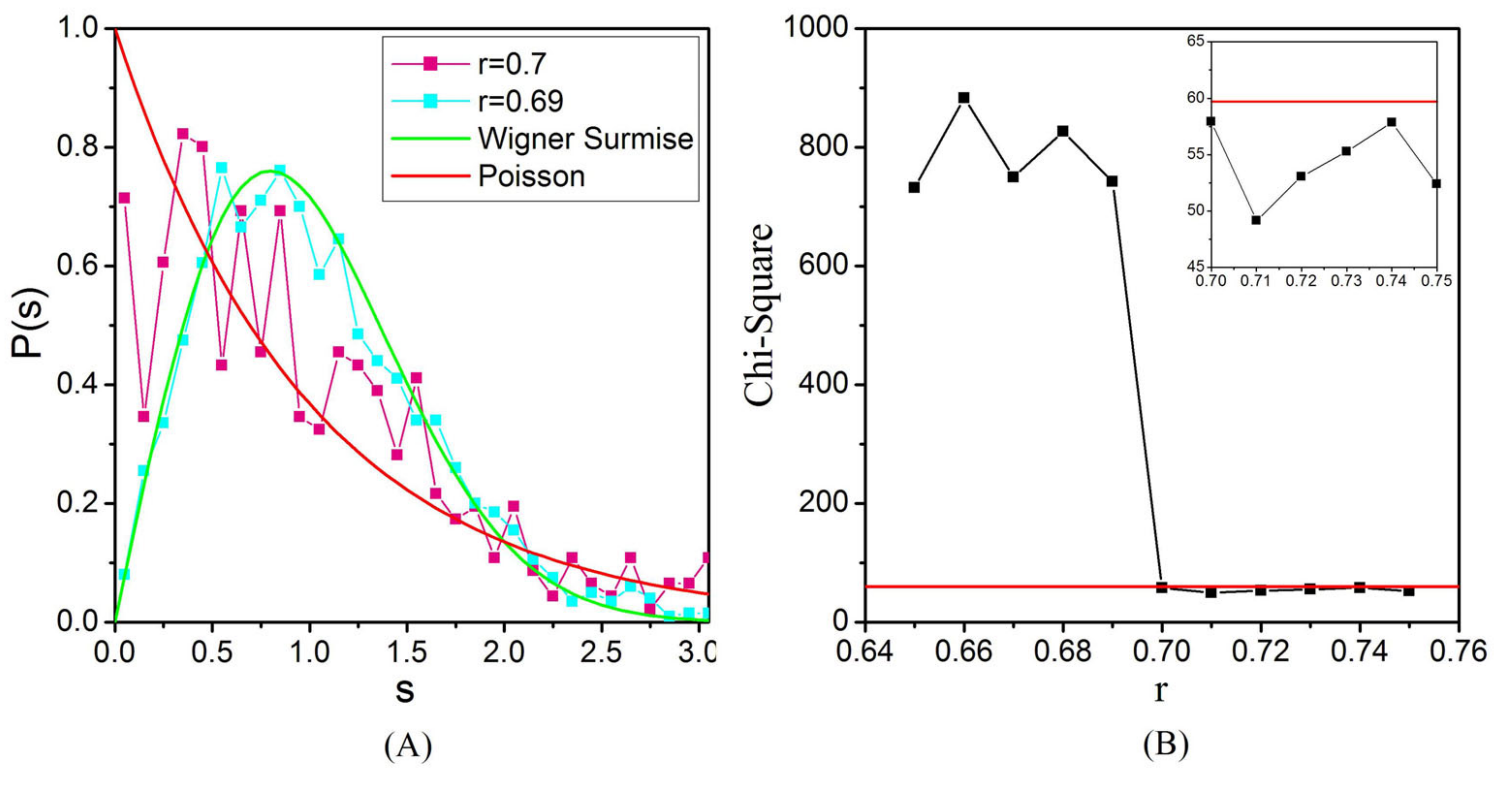
***In silico *evaluation of RMT approach**. An *in silico *modular system was constructed at cutoff of 0.7 to simulated a simplified gene co-expression network. (A) The normalized NNSDs of correlation matrices of the system at cutoff 0.69 (cyan) and 0.7 (pink) were compared to the curves of Wigner surmise (green) and Poisson distribution (red). The x-axis is the level spacing s and the y-axis is probability of NNSDs. (B) Chi-Square Test at different cutoff values. The red line in the inset indicates the critical value of Chi-Square test of *p *= 0.001. X and Y axes represent the cutoff and the chi-square test values, respectively.

To test the effectiveness of the RMT based criterion to determine correlation threshold for constructing co-expression network, we constructed an *in silico *model to simulate a simplified gene co-expression network. A correlation matrix of 2,000 genes with a designated correlation threshold of 0.7 was constructed (See Methods and Materials for details). The NNSD of the *in silico *correlation matrix showed a dramatic transition at the designated threshold (Fig. 1). NNSD followed Poisson distribution at the cutoff 0.7, whereas it obeyed GOE at cutoff 0.69. The RMT approach reliably identified the designated threshold.

### Sharp transitions of NNSD from GOE to Poisson distributions in correlation matrices from yeast microarray data

We then proceeded to apply RMT-based approach to real biological data. Yeast cell cycling microarray data [31] was selected because it has been extensively studied, making it easy to evaluate whether the results from RMT-based method are consistent with existing biological knowledge. A total of 5,293 genes with 70 time points available in the dataset were used. A correlation matrix based on pair-wise Pearson correlation coefficient in the range of (-1, 1) was calculated (See Methods for details). To simplify the analysis, the absolute cutoff values were set to be the same for both positive and negative correlations, though different cutoffs for positive and negative correlations were tested separately and similar results were obtained (data not shown).

**Figure 2:**
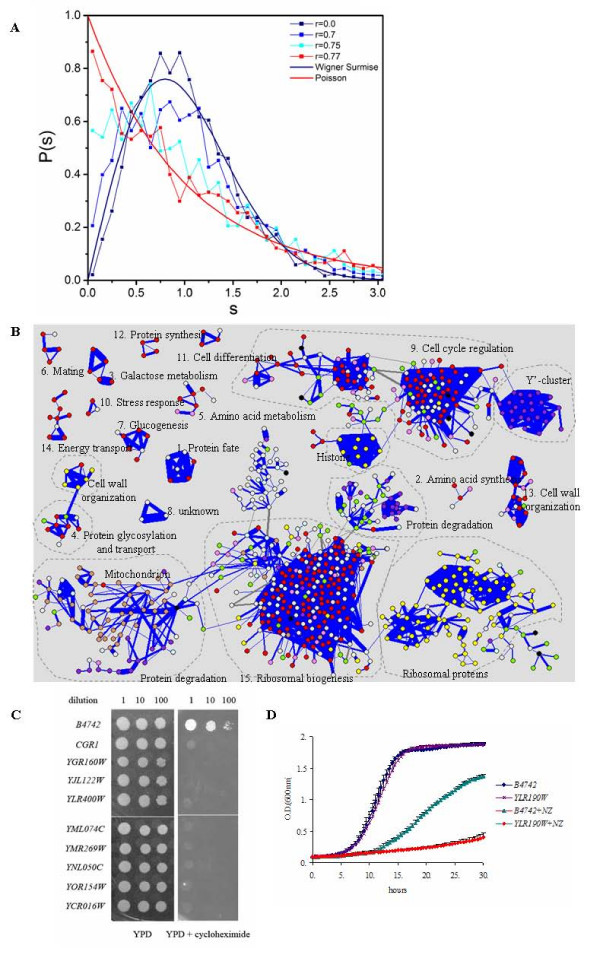
**Transition of nearest neighbour spacing distribution and gene co-expression network from yeast cell cycle microarray profiles**. (A) The normalized NNSDs of correlation matrices of yeast cell cycle gene expressions at different cutoff values. They were plotted against the curves of Wigner surmise (navy) and Poisson distribution (red). The x-axis is the level spacing s and the y-axis is the probability of NNSDs. (B) Fifteen significant gene co-expression sub-networks (modules) of the yeast cell cycling dataset were revealed at cutoff 0.77. All modules that have more than 4 genes are shown. For modules that have 3 genes, only those modules that form a cycle are shown, because only these kinds of modules are statistically significant [61]. Each node represents a gene and the width of line represents the Pearson correlation coefficient of two linked genes. Blue and gray lines indicate positive and negative correlation coefficients, respectively. Colors were assigned to nodes according to their functional categories: Red represents the major functional category of each module while purple, yellow and tan represent other functional categories, which are often clustered into sub-modules. Genes in lavender participate in processes closely related to genes in red. White nodes are unknown genes while black nodes are genes whose functional links to other genes are not currently understood. Green nodes are genes in metabolic processes, which are influenced by many biological processes. LightCyan nodes in Module 15 are genes involved in cell cycling regulation and related processes. Text in the map indicates the major functional category of each module, as represented by red. Dashed circles separate modules into sub-modules, which form independent modules at higher cutoffs. A more detailed description of each gene is provided in Additional File 3, Supplement Note B and online [62]. (C) Dilution assays of deletion mutants. Deletion mutants and the wild-type strain *B4742 *were grown in YPD overnight to saturation. Then cells were diluted 1:10 and 1:100 in water prior to spotting onto YPD or YPD containing 1 μg/ml cycloheximide plates. Images were obtained after incubation at 30°C for 4 or 7 days, respectively. (D) Growth curves of deletion mutant *YLR190W *and the parental strain *B4742*. Triplicates of *B4742 *and the *YLR190W *mutant were grown in YPD or YPD containing nocodazole (NZ) at 30°C with constant agitation for 30 hrs.

A clear sharp transition of NNSD from GOE to Poisson distribution was observed (Fig. 2A). Based on χ^2 ^test (*p *= 0.001), NNSD started to deviate from GOE at the correlation coefficient r_l _= 0.62 and completely transformed into Poisson distribution at the correlation coefficient r_h _= 0.77. Similarly, after the missing values is estimated using the nearest neighbour based method [32], sharp transition (see additional file 1, Figure 1) also observed and the threshold remains the same, which was possible due to the small number of missing values allowed in our study (only 7 missing values allowed in total 77 experiments). Furthermore, sharp transition from GOE to Poisson was also observed in correlation matrix using mutual information (data not shown). In addition, we applied this method to another yeast microarray dataset generated from environmental stress responses of yeast [33]. A clear transition from GOE to Poisson distributions was observed likewise (r = 0.60–0.89) (data not shown).

### Comparison of threshold obtained by RMT to that obtained by randomization

To evaluate the effectiveness of the threshold determined by RMT method, we compared it to the widely used method of determining the threshold by randomizing gene expression profiles [14,34], using the yeast cell cycle data [31] and environmental stress responses data [33]. Gene Ontology Slim category from *Saccharomyces *Genome Database (SGD) database [35] was used to classify links. To simplify the comparison, a link connecting two genes in the some Gene Ontology Slim category is deemed to be true. As summarized in Table 1, more than half of links in all networks obtained by RMT method are true links. However, for randomized method, the networks constructed from yeast environmental stress responses data contain very low percentage of true links. This comparison indicates that randomization is poor for certain microarrays and RMT method has an advantage over randomization in identifying system-specific information embedded inside microarray profiles.

**Table 1:** Comparison of thresholds obtained by RMT approach and randomization method and their corresponding co-expression networks. The thresholds determined by RMT approach and randomization method on two yeast microarray expression profiles and their corresponding co-expression networks are compared. Abbreviations: MI – mutual information, and Pearson – Pearson correlation coefficient.

Microarray data	Yeast cell cycle				Yeast environmental stress responses			
	RMT		Randomization		RMT		Randomization	

Correlation measure	MI	Pearson	MI	Pearson	MI	Pearson	MI	Pearson

r_h_	1.17	0.77	1.253065	0.68836	1.361	0.91	0.666697	0.533674
Number of genes	861	966	342	2398	402	523	3883	5266
Number of links	1764	3346	591	15853	4991	7160	625161	1585805
"true" links (%)	57.88	64.94	64.13	52.27	77.28	75.53	32.30	29.86

### Gene co-expression network based on yeast cell cycle microarray data

From correlation matrix of yeast cell cycle microarray data, we have constructed a co-expression network at the cutoff value r_h _= 0.77, where NNSD is completely transformed into Poisson distribution. The resulting network contains a total of 804 genes that are partitioned into 15 sub-networks (modules) (Fig. 2B; see Addtional File 3, Supplementary Note for detailed description of each module). Both positive and negative correlations are present in the network, as depicted in Figure 2B. To achieve an accurate evaluation based on current biological knowledge, we manually analyzed the biological coherence of modules according to gene annotations from the *Saccharomyces *Genome Database (SGD) and Munich Information centre for protein sequences (MIPS). Remarkably, all modules contain functionally coherent set of genes (Fig. 2B), demonstrating that RMT analysis faithfully reveals biologically meaningful networks in yeast. Indeed, among links of known genes, 85.4% of the links are between genes in the same or related functional pathways, whereas the rest 14.6% links are not supported by current experimental results, which might reflect the existence of systematic errors in microarray data or alternatively, the insufficiency of current biological knowledge of yeast.

Many large modules can be visually divided into smaller sub-modules, as indicated by the dashed circle in Figure 2B. For instance, Module 15 can be divided into four sub-modules: (1) ribosome proteins; (2) genes involved in ribosomal biogenesis; (3) mitochondrion proteins and (4) genes involved in protein degradation, while Module 9 contains distinct sub-modules of Y'-cluster genes, cell cycle regulators and histones. These have suggested the co-regulation at the gene expression level between different sub-modules, which often display evident functional association. For example, in Module 15, genes involved in ribosomal biogenesis are surely related to ribosome proteins, while a large portion of sub-module of mitochondrion proteins are indeed mitochondrion ribosome proteins. These results signify the presence of modular hierarchy in the network. Furthermore, sub-modules can be separated from each other by raising the cutoff. For instance, sub-modules of Module 15 are separated at cutoff of 0.79 (Additional File 1, Figure 2). Likewise, submodules of Module 9 are separated at cutoff of 0.79 and 0.81 (Additional File 1, Figure 2). Therefore, different levels of modularity of the yeast co-expression networks can be identified by further raising the cutoff values above the mathematically defined threshold.

Different types of modules are observed in the yeast co-expression network. Many (sub) modules are mainly comprised of components of protein complexes. Remarkable examples include the ribosomal protein sub-module in Module 15, in which about 90% of the known genes encode ribosomal or ribosome-associated proteins. Also, out of 10 genes of histone sub-module of Module 9, eight are histone subunits. In the case of mitochondrion, genes encoding proteins located to this small subcellular organelle are clustered into a sub-module of Module 15. In contrast, other modules cannot be classified by co-presence in protein complexes or subcellular organelles; instead, they are comprised of components in the same cellular processes. For instance, module 3 is composed of five genes participating in galactose metabolic pathway: *GAL1*, *2*, *3*, *7 *and *10*. Gal1p, Gal2p, Gap7p and Gal10p function in consecutive steps of glycolysis, whereas Gal3p is a regulatory protein exerting tight transcriptional control over the galactose metabolism pathway. These genes are co-regulated at expression level but might not interact with each other directly [36]. Similarly, all five known genes in Module 7 participate in gluconeogenesis, despite the lack of physical interaction between their protein counterparts.

It has been noted that genes with similar functions do not always have similar expression profiles [17]. Although two genes are not strongly correlated in gene expression, they could both be strongly correlated with the same set of other genes, a characteristic named as "transitivity". All of these transitive genes should be grouped together in the same modules. However, major clustering algorithms fail to do so [17]. In contrast, the co-expression network method is able to detect transitively co-regulated genes, as best exemplified by genes encoding ribosome proteins. The pairwise Pearson correlation coefficients between expressions of three genes encoding ribosome proteins, RPL19B, RPL26B and RPL1A, are less than 0.3 and hence unlikely to be grouped by clustering, whereas RMT analysis correctly links them within Module 15. Similarly, both Smc1p and Bim1p are involved in spindle formation or chromosome segregation during mitosis. Although the correlation between their expressions is as low as 0.2, they are grouped together by their linkages to other cell cycle regulators such as RAD53 and KCC4.

We also constructed the gene co-expression network from the yeast cell cycle microarray profiles with the missing values estimated [32]. As shown in Table 5, the two gene co-expression networks obtained from yeast cell cycle microarray profiles with or without missing value estimation are almost the same with 95.5% genes and 93.8% links overlapped.

**Table 5:** Comparison of gene co-expression networks obtained from yeast cell cycle microarray profiles with and without missing values estimation. The gene co-expression network obtained from original yeast cell cycle data is compared with the gene co-expression network obtained from a derived yeast cell cycle microarray data with missing value of original file is estimated by nearest neighbour method [32].

	Number of Genes	Number of Links
W/O	966	3346
With	941	3318
Overlap	922	3139

### Functional predictions of unknown genes and experimental validation

The fact that functionally related genes are connected together in the co-expression networks sheds the light for predicting the cellular roles of hypothetical genes by "guilt-by-association" [37]. Although confidence level of the predictions has not been quantified at this moment, it can be inferred by functional uniformity among the associated genes. We have tentatively predicted the functions of 136 genes based on yeast cell cycling datasets (see Additional File 2, Supplementary Table S1). A few selected examples are listed in Table 2. For example, yeast *YCR072C *is associated with many genes of ribosomal biogenesis and protein synthesis in Module 15. Accordingly, the protein product of this gene has been reported recently to participate in several complexes involved in protein synthesis and RNA turnover metabolism [38]. It was also co-purified with the 60S ribosomal subunit [39]. Notably, several predictions were consistent with experimental results but were not made by other network identification methods (Table 2).

To experimentally evaluate the predictive power of co-expression network, we examined the functional association of six unknown proteins (*YJL122W*, *YML074C*, *YMR269W*, *YNL050C*, *YOR154W*, and *YCR016W*) predicted to be involved in ribosomal biogenesis. While *YJL122W *and *YCR016W *were suggested to be involved in ribosomal biogenesis by other methods (Table 2), the other genes were not previously predicted to be associated with this functional process. Since we hypothesized that these genes participate in ribosomal biogenesis, we predicted that their deletion mutants might have defective ribosomes and deficiency in protein synthesis. Consequently, they should be sensitive to the protein synthesis inhibitor, cycloheximide. Indeed, a deletion mutant of *CGR1*, which is known to be involved in ribosomal biogenesis, failed to grow on YPD plates containing cycloheximide (Fig. 2D). Similarly, the deletion mutants of these six unknown genes, but not their parental strain *B4742*, failed to grow on YPD plates containing cycloheximide, indicating that protein synthesis in these mutants is defective. A possible function of ribosomal biogenesis of these unknown genes is also supported by recent high-throughput findings [40,41]. For example, *YML074C *and *YMR269W *localize to the nucleolus [40], the cellular organelle for ribosome biogenesis.

We also examined another unknown gene, *YLR190W*, for its role in cytokinesis. The deletion mutant and its parental strain *B4742 *grew similarly in YPD medium. However, the mutant showed a severe growth defect compared to *B4742 *in the presence of the cytokinesis inhibitor nocodazole (Fig. 2E). This has suggested that *YLR190W *is involved in cytokinesis. In conclusion, our experiments demonstrated the prediction power of the co-expression networks.

### RMT-based approach is applicable to microarray data of other tested organisms

**Figure 3:**
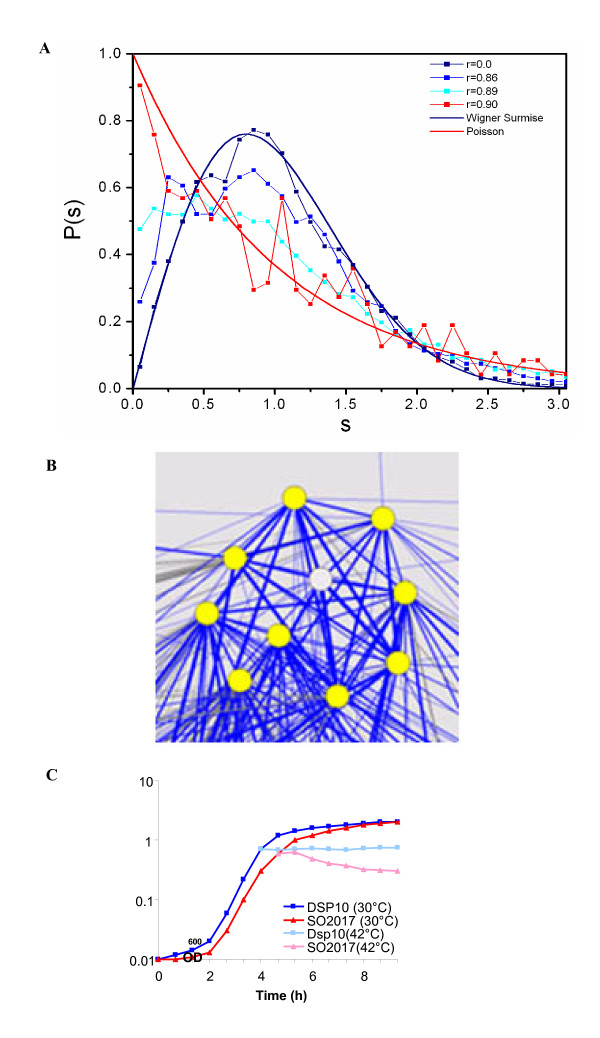
**Transition of nearest neighbour spacing distribution and gene co-expression network from *S. oneidensis *microarray profiles**. (A) The normalized NNSDs of correlation matrices of *S. oneidensis *heat/cold shock gene expressions at different cutoff values. They were plotted against the curves of Wigner surmise (navy) and Poisson distribution (red). The x-axis is the level spacing s and the y-axis is the probability of NNSDs. (B) A node representing a hypothetic protein SO2017 is interconnected to many heat shock proteins, suggesting a possible role in heat shock. The heat shock proteins are grpE, lon, SO3681, dnaJ, dnaK, groES, groEL, prlC and hslV. (C) Growth curves of SO2017 deletion mutant and its parental strain DSP10. Both strains were initially grown in LB media and shifted to 42°C when OD_600 _reached 0.69 and 0.62, respectively. In an independent experiment, the viability of the ΔSO2017 strain was reduced by 46% at 10 min after exposure to 42°C (data not shown).

The yeast co-expression network above was validated using existing gene annotations and experiments with deletion mutants for genes of unknown function. However, it is more desirable to apply the RMT-based method to determine correlation threshold for constructing gene co-expression networks from microarray data of little understood organisms. Since not much existing biological knowledge of these organisms could be employed to determine the threshold, it is appealing to use an automatic method to defined confident threshold. To test whether the NNSD transitions are present in correlation matrices of gene expression data from other organisms, the RMT method has been used to analyze correlation matrices of microarray data from a variety of organisms, e.g. *Shewanella oneidensis *[42], *Escherichia coli *[43], *Arabidopsis thaliana *[44], *Drosophila*[45], mouse [46] and human [47]. A clear NNSD transition from GOE to Poisson distribution has been observed in the range of 0.73–0.9 for the little understood bacterium *S. oneidensis *(Fig. 3A). Likewise, transitions have been revealed in other organisms: *E. coli *(r = 0.72–0.86), *A. thaliana *(r = 0.86–0.94), *Drosophila* (r = 0.76–0.93), mouse (r = 0.67–0.89) and human (r = 0.67–0.87) (data not shown). These results have demonstrated that RMT is applicable to gene expression data from all of the tested organisms.

The power of RMT method for determining the correlation threshold has been further evaluated in details based on genome-wide expression data from *S. oneidensis *and *E. coli*. A co-expression network of 7 modules was constructed for *S. oneidensis *heat/cold shock microarray data at cutoff of 0.90 (Additional File 1, Figure 3), and a co-expression network containing 30 modules was identified in *E. coli *dataset at a cutoff of 0.86 (Additional File 1, Figure 4). Similar to yeast data, functional modules was identified in the networks [62]. For example, modules of energy transport (#1, 2 and 4) were isolated in *S. oneidensis *co-expression network, while the big module #25 in *E. coli *dataset was dedicated to display sugar to the outer structure of bacterial surface. Taken together, the RMT-approach is useful to determine the correlation threshold for identifying gene co-expression networks in different species.

**Figure 4:**
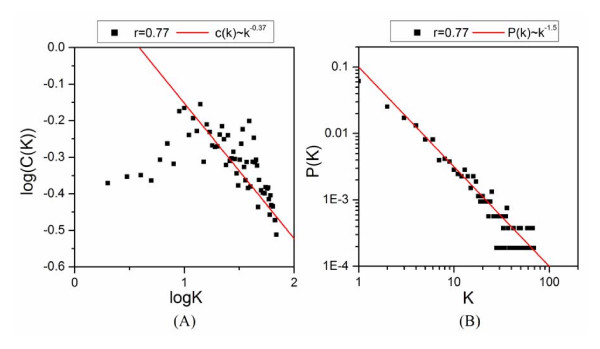
**Structural properties of gene co-expression network from yeast cell cycling data at threshold of 0.77**. (A) The dependence of the clustering coefficient on the node's degree *C*(*K*). (B) The connectivity distribution *P*(*K*).

From the co-expression networks, we predicted functions of 32 unknown genes for *E. coli *and 47 unknown genes for *S. oneidensis * (see Additional File 2, Supplementary Tables S2 and S3). Some representative examples are listed in Table 2. For example, *E. coli *gene *yaeC *is predicted to function in metabolite transport. This prediction is supported by a recent report that *yaeC *is a component of the methionine uptake system [48], though this information is missing in the annotation database from the institute for genomic research (TIGR). The predictive power of co-expression network was further experimentally tested on a hypothetical protein SO2107 from *S. oneidensis*. It formed a compact sub-network with other known heat shock proteins, indicating a role of this gene in heat shock response (Fig. 2B). An in-frame deletion mutant was generated, and it was indeed sensitive to heat shock (Fig. 3C). In addition, an RpoH (σ^32^) binding site was identified in the upstream region of SO2017 [42]. These results clearly supported the reliability of the gene function predictions made using co-expression network.

**Table 2:** Representative functional predictions of hypothetical proteins with high confidence.

**Species**	**Module**	**Gene Designation**	**Predicted biological pathway/localization**	**Identified by other methods?**	**Experimental verification in this paper**	**Experimental supporting evidences from annotation databases**
**Yeast**	Module 9	*YLR183C*	cell cycle	yes [19, 63]	ND	transcription factor regulating several promoters of genes involved in pheromone response and cell cycle;
	Module 9	*YOL007C*	cell cycle	yes [19, 63]	ND	structural component of the chitin synthase 3 complex
	Module 9	*YLR190W*	cytokinesis	yes [19]	Consistent with prediction	localized to small buds, bud neck, and incipient bud sites; mRNA is targeted to the bud via the mRNA transport system involving She2p
	Module 9	*YNL058C*	cell cycle	yes [18, 19]	ND	potential Cdc28p substrate
	Module 15	*YHL021C*	Stress response	yes [19, 63]	ND	
	Module 15	*YGR160W*	ribosome biogenesis	yes [19, 63]	Consistent with prediction	
	Module 15	*YIL127C*	ribosome biogenesis	yes [63]	ND	
	Module 15	*YJL122W*	ribosome biogenesis	yes [63]	Consistent with prediction	
	Module 15	*YLR196W*	ribosome biogenesis	yes [19, 63]	ND	nucleolar protein
	Module 15	*YLR400W*	ribosome biogenesis	no	Consistent with prediction	
	Module 15	*YML074C*	ribosome biogenesis	yes [19]	Consistent with prediction	nucleolar peptidyl-prolyl cis-trans isomerase (PPIase); FK506 binding protein; phosphorylated by casein kinase II (Cka1p-Cka2p-Ckb1p-Ckb2p) and dephosphorylated by Ptp1p
	Module 15	*YMR269W*	ribosome biogenesis	no	Consistent with prediction	nucleolar protein [40]; protein possibly involved in protein synthesis [64]
	Module 15	*YNL050C*	ribosome biogenesis	no	Consistent with prediction	
	Module 15	*YOR146W*	ribosome biogenesis	no	ND	
	Module 15	*YOR154W*	ribosome biogenesis	no	Consistent with prediction	
	Module 15	*YCR016W*	ribosome biogenesis	yes [63]	Consistent with prediction	nucleolar protein
	Module 15	*YPR169W*	ribosome biogenesis	no	ND	nucleolar protein [40]
	Module 15	YCR072C	ribosome biogenesis	yes [19, 63]	ND	present in several complexes involved in protein synthesis and RNA turnover metabolism [38]; co-purified with the 60S ribosomal subunit [39].
	Module 15	*YDL063C*	ribosome biogenesis	yes [19]	ND	
***S. oneidensis***	Module 4	SO3725	central intermediate metabolism; protein modification	no	ND	
	Module 5	SO2017	heat shcok response	yes [42].	Consistent with prediction	
	Module 5	SO2042	heat shcok response			
	Module 5	SO2375	metabolism			
	Module 5	SO3298	metabolism	no	ND	
	Module 5	DsbD	metabolism	no	ND	thiol:disulfide interchange protein
***E. c***	Module 143	yaeC	metabolism, cell surface transporter	no	ND	
	Module 143	ybgF	protein synthesis	no	ND	
	Module 141	envR	function related to cell surface structure	no	ND	
	Module 2531	b1505	transport protein	no	ND	
	Module 2531	yqcB	energy metabolism	no	ND	

The function of the listed hypothetical proteins was not clearly understood. However, many of our predictions were supported by existing experimental evidence from other laboratories (see last column), which was cited and summarized in the *Saccharomyces *Genome Database or TIGR's *S. oneidensis *or *E. coli *annotation database [59].

ND: not determined.

**Table 3:** Effect of different *Ct *value on sampling to recover 99% of "true" links for a system of 2000 genes

*Ct*	0.3	0.5	0.7
Number of sampled expressions	600	200	50

### Topological properties of gene co-expression networks

Biological networks are considered to be small world, modular, hierarchical and scale-free [3,49]. To determine whether the obtained co-expression networks are consistent with general network theory, the topological properties of the co-expression network from yeast cell cycle microarray data have been examined. The average path length of this network is 7.81, which is quite small compared to the size of the network (804 genes). This result suggests that the network is a small world. The average clustering coefficient of this network is 0.323, implying a high degree of modularity. Also, the average clustering coefficient (C(k)) of all genes with k links follows the scaling law: C(k) ~ k^-0.37 ^as shown in Figure 4A. This signifies high hierarchical modularity although the scaling exponent of 0.37 differs from the values obtained from metabolic modular networks [50]. Analysis of connectivity properties of this network revealed a power-law distribution with a degree exponent of 1.5 (Fig. 4B), which is in accordance with the previous results on microarray expression profiles [51]. Taken together, the properties of RMT networks are consistent with general network theory.

### Computational evaluations of RMT approach

**Figure 5:**
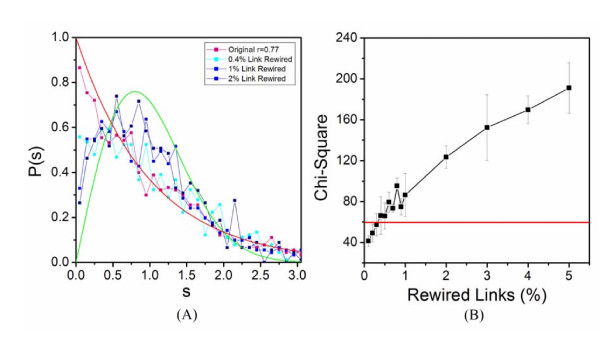
**Sensitivity of RMT approach**. (A) Wigner surmise (green) and Poisson distribution (red) were compared to the normalized NNSDs of the yeast dataset at cutoff 0.77 and its derived correlation matrices in which 1% (cyan), 2% (blue), 3% (navy) links were rewired. (B) Chi-Square test. Poisson distribution is plotted against NNSDs of correlation matrix of yeast cell cycling dataset at cutoff 0.77 and its derived correlation matrices in which 0.1%, 0.2%, 0.3%, 0.4%, 0.5%, 0.6%, 0.7%, 0.8%, 0.9%, 1%, 2%, 3%, 4%, 5% links are rewired. The red line indicates the critical value of Chi-Square test of *p *= 0.001.

**Figure 6:**
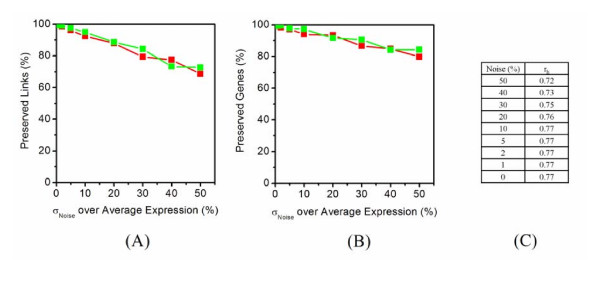
**Analysis of RMT approach for robustness to noise**. Increasing levels of Gaussian noise are added to the yeast cell cycling microarray expression profiles. The mean of noise is zero and its standard deviation (σ_Noise_) is set to 1, 2, 5, 10, 20, 30, 40, and 50% of the average of absolute expression value of whole dataset. (A) Percentage of preserved links over total links in the modules perturbed by noise (red), and over total links in original modules (green) at different levels of noise. (B) Percentage of preserved Genes over total genes in the modules perturbed by noise (red), and over total genes in original modules (green) at different levels of noise. (C) Increased noise decreases the cutoff to separate modules.

To determine the sensitivity of RMT-based approach to determine correlation threshold for identifying gene co-expression networks, we randomly have rewired a small percentage of the links in the network from the yeast cell cycling data. As low as 0.4% random rewiring is able to make the NNSD deviating from Poisson distribution (Fig. 5). Therefore, the RMT approach is sensitive to detect even small topological changes in the networks. In addition, since microarray data typically contain high inherent variability, we have examined whether the networks are stable when additional noise is added. Different levels (1–50%) of Gaussian noise have been added to the entire dataset; new correlation thresholds have been determined for the perturbed data and corresponding networks have been constructed. When 30% noise has been added, 79.4% of the original links and 86.5% of the original genes are still preserved (Fig. 6), indicating that the RMT approach is robust in tolerating noises. Together, these statistical evaluations have indicated that the RMT approach is sensitive and robust to noise for determining correlation threshold.

### *In silico *evaluation of the effect of sampling complexity on RMT approach

The microarray data is under-sampled, that is, the number of experiments is fewer than the number of genes. To evaluate the effect of under-sampling to RMT based method, we developed an *in silico *modular gene system (see Methods and Materials for details), in which correlations between expressions of genes inside modules ("true" links) have been designed to be a value *Ct *(0 <*Ct *< 1); and other expression correlations have been designed to be zero. Then, RMT based method has applied to construct gene co-expression network from sampled expression data of the modular system. First, effect of different *Ct *value on the number of samplings to recover 99% of "true" links is tested. As shown in Table 3, when the *Ct *value is decreased (from 0.7 to 0.3), the number of expressions needed to separate the "true" links from random is increased (from 50 to 600). However, the number of experiments is still very small comparing to the total genes in the system. In addition, the effect of the size of system on the number of samplings needed is limited (Table 4). We also examined the percentage of "true" links over total links in the network. For *Ct *= 0.7 and a system size of 2000 genes, the percentage of "true" links over total links is 98.4% for only 20 expressions sampled (data not shown). In summary, even with the under-sampled expressions, the RMT approach is still able to recover the original co-expression networks in the designed model.

**Table 4:** Effect of the size of gene systems on sampling to recover 99% of "true" links for certain *Ct *= 0.7.

Size of gene system	2000	3000	5000	7000
Number of sampled expressions	50	70	100	100

## Discussion

RMT has been used in characterizing the non-random phenomena in physical, material and social systems, including heavy nuclei, metal insulator transitions and the stock markets. It has been well recognized in these systems that RMT analyses are efficient for distinguishing system-specific, nonrandom properties from random noise. In this study, our observations with the microarray data from various organisms support our central hypothesis that the two universal predictions are applicable to biological systems. RMT might be particularly suitable for microarray data, which usually have high inherent variations. Based on yeast gene annotation, 85% of the functionally known genes are correctly linked. In addition, we demonstrated that the structure of network obtained from original microarray profiles differs from the networks obtained from randomized expression data [52]. Furthermore, computational analysis showed that all modules and links in an *in silico *network were correctly identified at the expected cutoff value. Together, these results suggest that the RMT-based approach can reliably identify gene co-expression networks.

Previously, we have applied the random matrix theory to study the properties of microarray data [50] and biological networks [54]. In our Physics Letters A paper [54], we have demonstrated that the NNSDs of the adjacent matrix of protein interaction network and metabolic network follow universal predictions of RMT. The current manuscript is a follow-on study of our Physical Review E paper [50]. The Physical Review E paper has just shown that NNSDs of correlation matrices from microarray data follow the universal description in random matrix theory. However, it did not provide a solid and complete algorithm for inferring gene co-expression networks from microarray data. Moreover, no rigorous biological tests of the predicted networks were performed. In the current manuscript, we systematically proposed a method for inferring gene co-expression network by utilizing the transition of NNSDs of correlation matrices from microarray data. And we provided computational analyses to show that this approach is reliable, sensitive and robust to noise. Furthermore, we demonstrated that the resulting co-expression networks are biologically meaningful. We provided evidences that the gene grouped in the network participate in the same biological pathway; the function of unknown genes can be accurately predicted, as shown by the experimentally validation; and the network are hierarchical, modular, small-world and scale-free, which are typical properties of biological networks. Together, the current manuscript is dedicated to a more practical method for inferring biological meaningful gene networks from microarray data, which is certainly not tackled at all in the previous publication. The current manuscript is a necessary follow-on study and also the first manuscript to provide a useful RMT based method for systems biology to identify biological pathways that are regulated by the given condition, to annotate function of unknown genes, and to dissect the global network properties.

There are two kinds of properties of eigenvalues of a real symmetric matrix: global properties and local properties. For example, eigenvalue distribution that changes based on large scale of eigenvalues is a global property of eigenvalues [23]. On the other hand, NNSD is a local property of eigenvalues. The global and local properties usually are unrelated. Numerical experiments showed that local properties of eigenvalues of a real symmetric matrix become independent of the probability distribution of matrix elements and the global properties of matrix when N → ∞ [23]. The local properties, like NNSD, only dependent on over-all symmetries of the system, like real symmetric, or Hermitian.

As a matter of fact, the correlation matrices of microarray data are not general matrices with random elements, or even normal correlation matrices in statistic due to the number of microarray experiments is much less than the number of genes analyzed. Hence, current predictions of RMT about the global properties, such as eigenvalue distribution, may be invalid for these correlation matrices from microarray data. Additional File 1, Figure 5 has showed that the distribution of elements in correlation matrix of yeast cell cycle microarray data follows the Gaussian distribution and Additional File 1, Figure 6 has showed that the eigenvalue distribution of this matrix follows Cauchy distribution. However, the overall symmetry of these correlation matrices is still a real symmetry. The independent character of NNSD makes it possible to compare the NNSD of correlation matrices from microarray profiles with the theoretical predictions of RMT. Noted that the correlation matrix used in the report actually even is not a normal semi-positively defined correlation matrix as there are missing values in the data set and we have only used the experiments both genes having values to calculate the correlation. However, the same NNSD transitions have also been observed on normal semi-positively defined correlation matrices that calculated from same microarray data after estimating the missing value [32].

The co-expression networks identified based on RMT criterion can serve as a useful tool for predicting functions of hypothetical proteins. Genome sequencing projects indicate that substantial portions of open reading frames in a variety of organisms are functionally unknown. Defining the functions of such genes is a formidable task [53]. In this study, the cellular roles of 215 functionally unknown genes from yeast, *S. oneidensis *and *E. coli *were predicted, many of which were supported by existing information and our experiments. Such predictions will provide directions and guidance for future experimental design and verification, and hence facilitate the studies of functionally poorly characterized genes. In addition, the co-expression networks appear to be fairly sensitive in identifying functional modules, as shown by our computational analyses and comparison to relevance network. Indeed, the functions of several unknown genes identified and experimentally verified in this report were not reported before by other commonly used methods including relevance network.

We selected the threshold at which the NNSD finished the transformation into Poisson distribution from GOE distribution with the probability of *p *= 0.001. Based on the microarray data examined, it appears that the sharpest changes of χ^2 ^values were observed when NNSD is changed to a Poisson distribution at the probability level of *p *= 0.001. Thus *p *= 0.001 appears to be a good choice. However, due to the nature of microarray data (e.g. high noise), insufficient datasets to resolve the interactive relationships among different genes and/or the complexity of biological processes, complete removal of noises is unlikely. It is expected that some false links could still exist above the threshold. To further remove false links, one could select the threshold at other correlation values at which the NNSD is changed into a Poisson distribution with different probabilities such as *p *= 0.01, 0.05, or 0.1, which correspond to higher threshold correlation values. This should enable the isolation of network connections with higher confidence and further division of larger modules into smaller modules.

Our results have indicated that a transition zone can be defined based on the two critical points (r_l _and r_h_). A transition zone of r = 0.62–0.77 has been identified for yeast cell cycling data. In order to achieve high confidence on the co-expression network, we have chosen the upper bound of the NNSD transition as the threshold, with trade-off of possible loss of some correct information. For instance, at the threshold value of r = 0.77, a module involved in galactose metabolism has been identified (Module 3). But a key regulatory protein in this pathway, Gal80, is missing. This protein has been identified after lowering threshold value to 0.70 (data not shown). Therefore, lowering the threshold in the transition zone can enable us to identify additional correct links. However, this could also lead to much more false links (data not shown). One solution to solve this problem is called soft thresholding [15]. A further study that combines RMT based criterion with soft threshold to identify more true links in the transition zone will be worthy of investigation in the future.

When Mc can not be easily distinguished from Mr in correlation matrix, the RMT approach could not be able to generate a meaningful threshold. The examples include occasions that the experiment points are very limited and similarities between expressions of all genes are high; or very messy microarrays that have been poorly carried out.

## Conclusion

Although high throughput genomics technologies such as microarray are powerful tools for studying gene functions and global regulations, identifying cellular networks in an automatic and objective fashion from genome-wide gene expression data remains challenging [6,19,54]. The RMT-based approach has been presented here provides a reliable, sensitive and robust method for determining correlation threshold; and then dissecting gene co-expression networks. The automatic and objective fashion of RMT-based approach makes it more advantageous in studying little understood organisms. The RMT-based approach could also be applied to other high throughput data for proteomes and metabolomes or combinations of these datasets. Similar characteristics have been also observed with the matrices from yeast protein interaction and metabolic pathway data [55]. Moreover, we expect that RMT is applicable to complex biological systems such as communities, and ecosystems. Further exploration of this method should provide valuable insights into the modular networks across different levels of biological organization.

## Methods

### Determining correlation threshold by RMT based approach

First, a gene expression correlation matrix M, whose elements are Pair-wise Pearson correlation coefficients (r) in the range of (-1.0, 1.0), was constructed. If there are missing values in the expression files, only the experiments that both genes have values are used to calculate Pearson correlation. Then, a series of correlation matrices were constructed using different cutoff values. If the absolute value of an element in the original correlation matrix is less than the selected cutoff, it is set to 0. Eigenvalues of each correlation matrix were obtained by direct diagonalization of the matrix. Standard spectral unfolding techniques [26] were applied to have a constant density of eigenvalues and subsequently the nearest neighbour spacing distribution P(s) (see Additional File 3, Supplemental Note A for details), which is employed to describe the fluctuation of eigenvalues of the correlation matrix. We used the Chi square test to determine two critical threshold values, r_l _at which P(s) start to deviate from GOE at a confidence level of *p *= 0.001, and r_h _at which P(s) follows the Poisson distribution at a confidence level of *p *= 0.001. The critical point r_h _is chosen to be the threshold used for constructing the gene co-expression network. Same procedure is used for analyzing the correlation matrix based on mutual information to determine the threshold.

### Constructing gene co-expression network

Based on RMT, the complete transition from GOE to Poisson distributions can serve as a reference point to distinguish system-specific, nonrandom properties embedded in gene expression data from random noise. Thus, we used the r_h _at which NNSD is completely transformed into Poisson distribution at the significance level of *p *= 0.001 as the threshold to define gene co-expression networks. From the correlation matrix, we can easily construct a co-expression network, in which each gene is a node and there is a link between two genes if the correlation measure between their expressions is greater than the threshold. All co-expression networks with cutoff beyond threshold will also provide system-specific relationship. However, the co-expression network at the threshold will provide the most relationship.

### Construction of an *in silico *model

An *in silico *gene co-expression network of 2,000 genes with 10 modules of the size ranging from 5 to 200 genes (200, 100, 50, 50, 20, 20, 10, 10, 5, and 5 genes) was constructed using the threshold value of r_h _= 0.7. The corresponding *in silico *correlation matrix was constructed as following: First, for each module whose size is greater than 5, we assigned the correlation coefficient of a random value in the range of +/-(0.7, 1.0) to each pair of element inside the module with a probability of 0.35. This probability is chosen to make the modules have the similar number of "true" links as the real gene co-expression modules. Second, for two modules with a size of 5, we linked the 5 elements to form some topology structure similar in real gene co-expression networks by assigning certain correlation coefficient with a random value in the range of +/-(0.7, 1.0). Finally, we assigned the rest correlation coefficient in the correlation matrix of these 2000 elements to a random value in the range of (-0.695, 0.695). The links with weight between 0.7 and 1.0 correspond to the "true" correlation inside modules and the links with weight between 0.0 and 0.7 are noise. The RMT method was applied to this system as described above.

### Sampling expressions of an in silico modular gene system

A modular gene system has been developed to examine the effect of under-sampling on RMT based method. In this gene system (with size > 1000), there are 1000 genes divided into ten modules with equal size. Correlations between expressions of genes inside modules ("true" links) have been designed to a value *Ct *(0 <*Ct *< 1); and other expression correlations have been designed to be zero. Expressions of genes are sampled from Gaussian distribution to make their correlation matrix following previous designed pattern. Note that the real correlations from the sampled expression will fluctuate around the original designed values. Then, RMT based method was applied to sampled expressions to construct gene co-expression network. Multiple sampling experiments have been conducted. First, for a system of 2000 genes and different *Ct *value (0.3, 0.5, and 0.7), we started from sampling 20 expressions, and then sampled 10 more expressions every next time until 99% of "true" links recovered. Second, for certain *Ct *value (0.7) and different sizes of system (2000, 3000, 5000, 7000), we started from sampling 20 expressions, and then 10 more expressions every next time until 99% of "true" links recovered.

### Evaluations of RMT-based network identification approach

**(A) Sensitivity**. The Maslov-Sneppen procedure [56] was used to rewire a small percentage (0.1%, 0.2%, 0.3%, 0.4%, 0.5%, 0.6%, 0.7%, 0.8%, 0.9%, 1%, 2%, 3%, 4%, 5%) of links in the gene co-expression network derived from yeast cell cycling data. The Maslov-Sneppen procedure randomizes the links in the network while keeping the degree of each vertex unchanged. For each rewiring, two links, A-B and C-D, are randomly selected. If there is no link between A and D and no link between C and B, the original two links A-B and C-D are disconnected and two new links A-D and B-C are constructed. After finishing rewiring the network, we constructed the corresponding correlation matrices, from which NNSDs were obtained. **(B) Robustness to noise**. Increasing levels of Gaussian noise (1–50%) were added to the entire yeast cell cycling data. Then NNSDs from perturbed data were calculated as described above.

### Network visualization and annotation

The software program Pajek [57] was used to visualize modular networks. Annotation databases for yeast, *S. oneidensis *and *E. coli *were from the *Saccharomyces *Genome Database (SGD) [58] and The Institute for Genomic Research [59], respectively.

### Strains and physiological studies

All yeast strains were purchased from Open Biosystems (Huntsville, AL). For dilution assays, cultures were grown in YPD media (1% yeast extract, 2% bactopeptone, and 2% dextrose) overnight to stationary phase, suspended in water, and then spotted onto chemical-containing YPD plates at 10-fold serial dilutions followed by incubation at 30°C. Growth assay of cultures in liquid medium was performed in YPD with a BioScreen C (MTX Lab Systems, Inc., Vienna, VA).

To generate an in-frame *S. oneidensis *mutant of SO2017, the majority of the ORF was removed by an in-frame deletion mutagenesis approach [60]. The desired gene region was removed using PCR amplification with the primers A1 (5'AGC CTG TGA GCT CAC GGG), A2 (5'TGT TTA AAC TTA GTG GAT GGG GGT TAG ATC GAG GAT ATT), B1 (5'CCC ATC CAC TAA GTT TAA ACA GTT TGG CAA ACC AAT ATC) and B2 (5'ACA ATC GAG CTC TGC GAT), and a second cross-over PCR amplification with A1 and B2 using the mixed amplified fragments as templates. The resulting product was cloned into the suicide plasmid pDS3.0 and transformed into *E. coli *S17-1/λ_*pir *_prior to conjugal transfer into *S. oneidensis *strain DSP10. Correct in-frame deletion was verified by DNA sequencing.

Growth of the *SO2017 *mutant was examined in Luria-Bertani (LB) medium under both optimal temperature (30°C) and temperature shifting (30 to 42°C at OD_600 _= 0.62). The survival of mutant cells was compared to the parental strain DSP10 at 5, 10, 15, 25, 50 min after temperature shift.

## Authors' contributions

JZZH and JXZH developed the original ideas.

FL and JXZH: Develop and implement the algorithms and computational evaluation.

FL and YY: Analysis the gene networks.

YY and HG: Design and conduct the biological experiments.

FL, YY, JXZH and JZZH: writing the manuscript.

All the other contributed to the read and approved the final version.

## Supplementary Material

Additional file 1 Figures showed the co-expression network constructed from microarray profiles of yeast, *S. oneidensis *and *E. Coli*. Click here for file

Additional file 2 Notes for the understanding of random matrix theory and the description of modular networks obtained from microarray profiles of yeast, *S. oneidensis *and *E. Coli*. Click here for file

Additional file 3 Tables showed the 215 functionally unknown genes from yeast, *E. coli *and *S. oneidensis *are predicted by the gene co-expression networks using guilt-by-association principle. Click here for file
