# Supplementary material for: Constructing gene co-expression networks and predicting functions of unknown genes by random matrix theory
Source: BMC Bioinformatics. 2007 Aug 14;8:299. doi: 10.1186/1471-2105-8-299 (PMC2212665; doi:10.1186/1471-2105-8-299)
Supplement: Additional file 1 — Figures showed the co-expression network constructed from microarray profiles of yeast, S. oneidensis and E. Coli. [file 1471-2105-8-299-S1.pdf]

SUPPLEMENTAL FIGURES

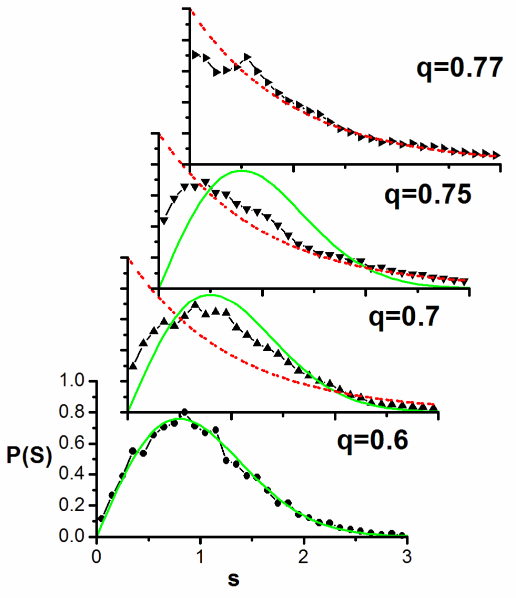

Supplemental Figure 1. The normalized NNSDs of correlation matrices of missing value estimated yeast cell cycle microarray data at different cutoff values.

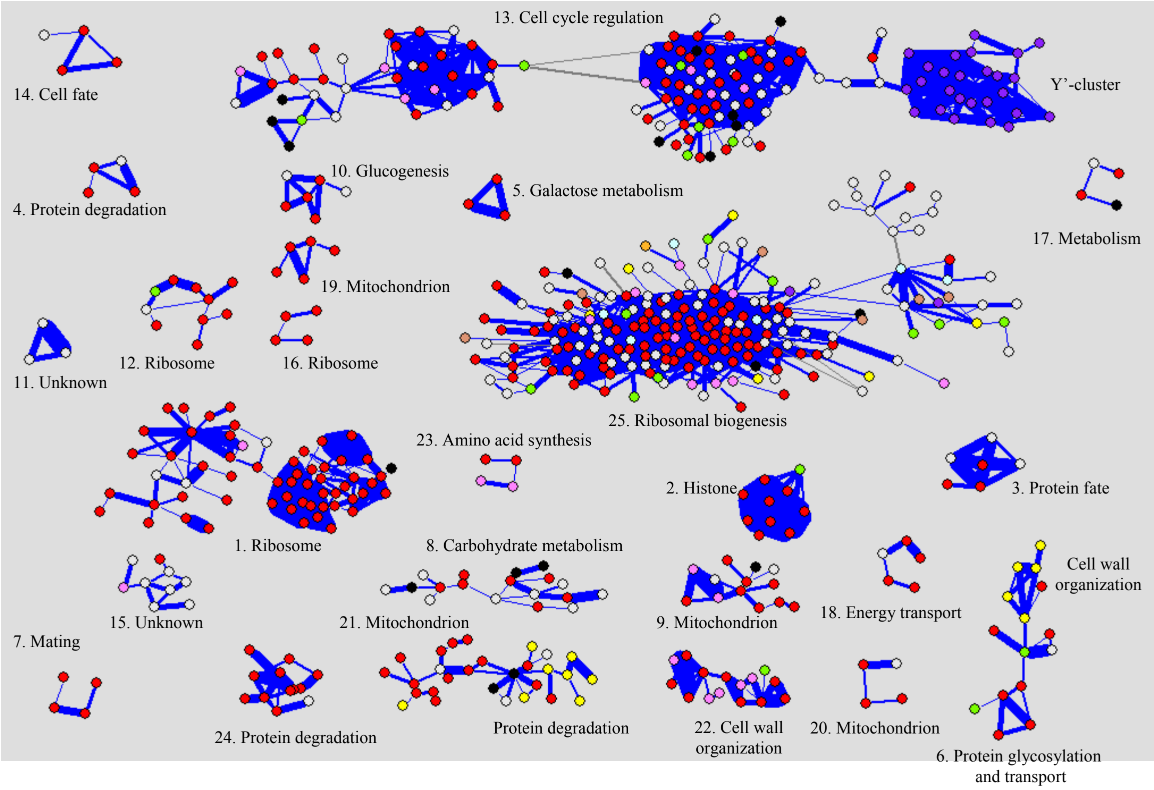

Supplemental Figure 2. Raising cutoff to 0.79 further disintegrates 15 modules of cutoff 0.77 into 25 smaller modules. Sub-modules at cutoff 0.77, as indicated by dashed circles, become independent module(s) at 0.79 or higher cutoffs (data not shown). Colors were assigned in the same way as Figure 2B. Blue and gray lines indicate positive and negative correlation coefficients, respectively.

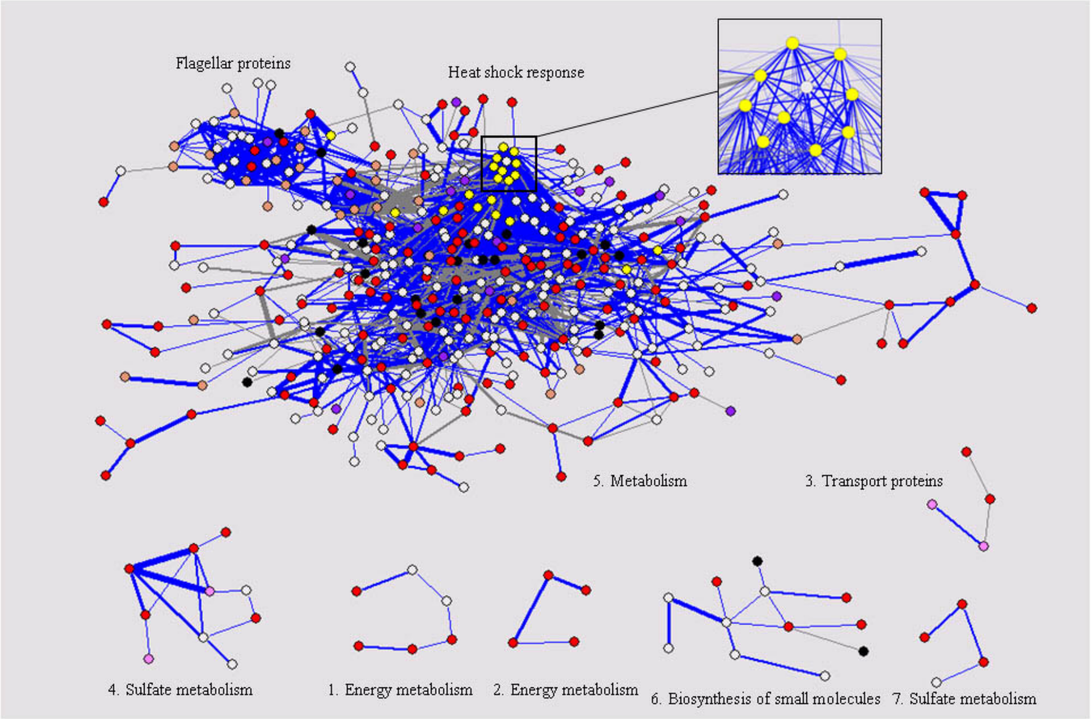

Supplemental Figure 3. Seven modules were isolated from the *Shewanella* data. Colors were assigned in the same way as Figure 2B. Detailed description of each gene is shown in Supplement Note C and online from http://www.cs.clemson.edu/~luofeng/RMT.html. The inset indicated is presented as Fig. 3B in the text.

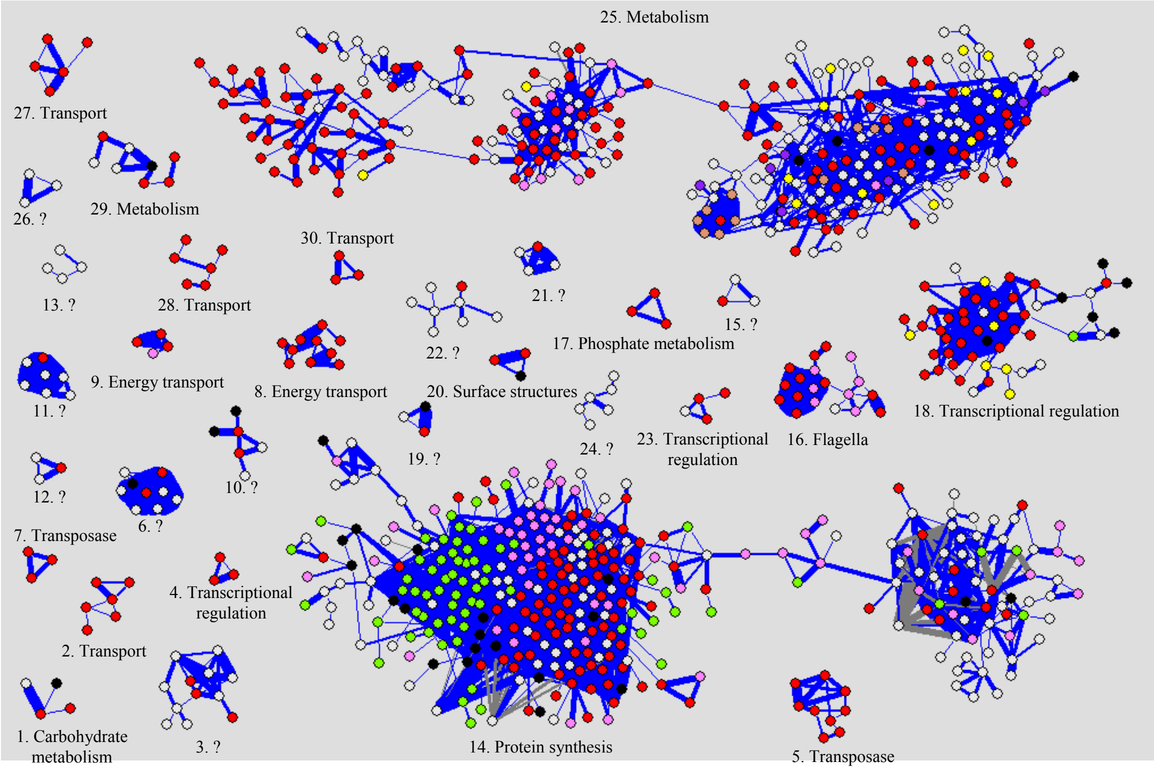

Supplemental Figure 4. Thirty modules were isolated from the *E. coli* dataset at cutoff of 0.86. Colors were assigned in the same way as Figure 2B. Blue and gray lines indicate positive and negative correlation coefficients, respectively. Detailed description of each gene is shown in Supplement Note D and online from http://www.cs.clemson.edu/~luofeng/RMT.html.

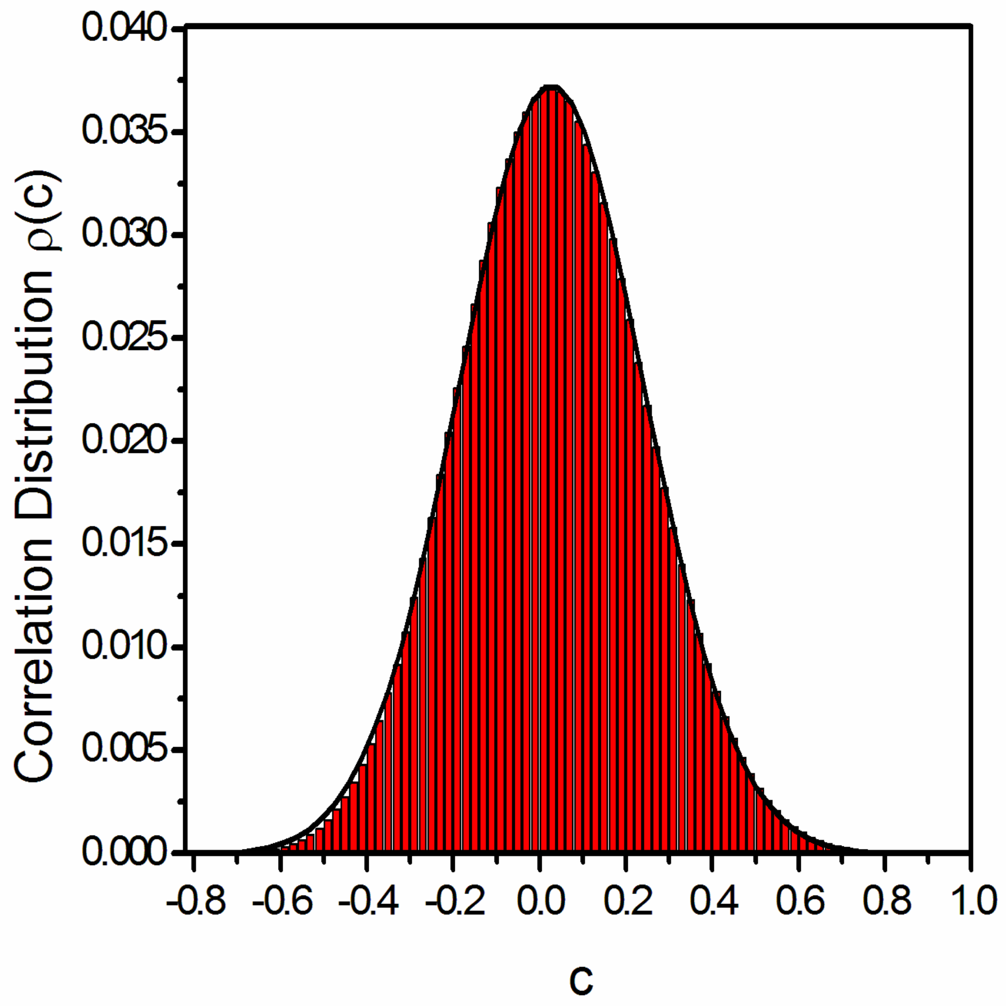

Supplemental Figure 5. The distribution of elements of correlation matrix calculated from yeast cell cycle microarray data (See methods). The black line is the Gaussian fit of the distribution.

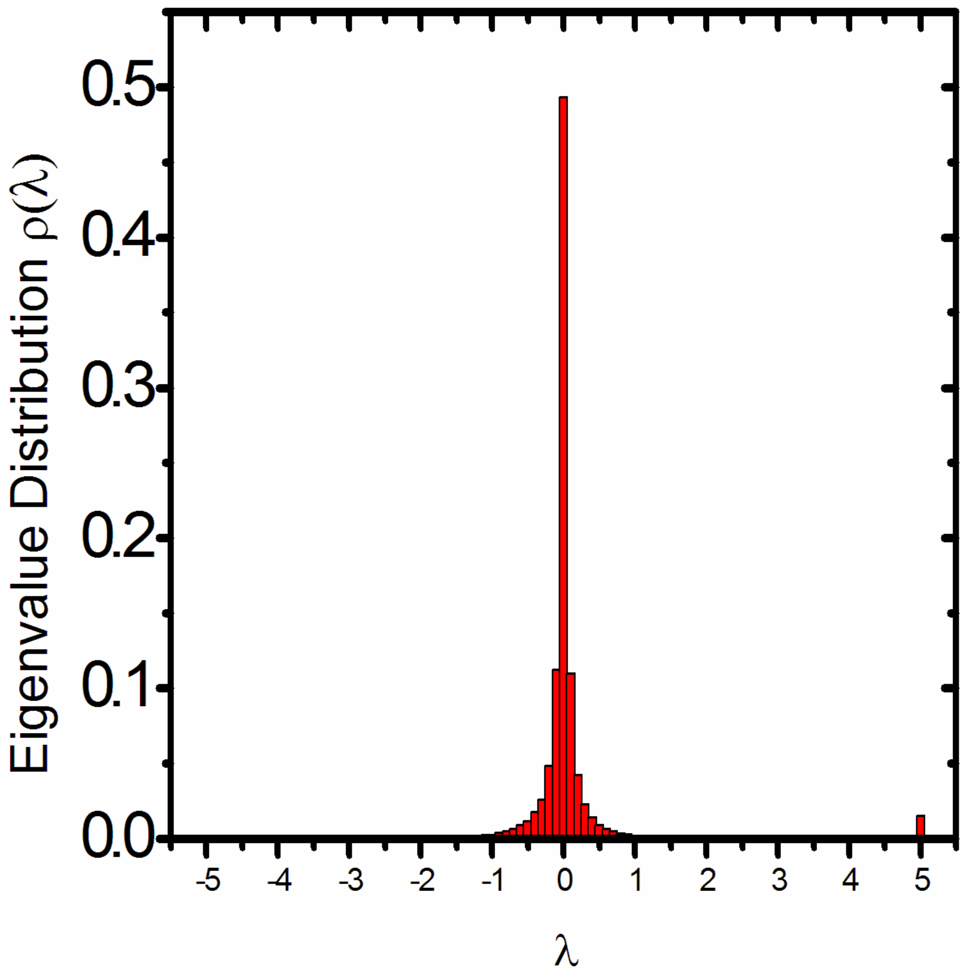

Supplemental Figure 6. The distribution of eigenvalues of correlation matrix calculated from yeast cell cycle microarray data (See methods).
