# Supplementary material for: Constructing gene co-expression networks and predicting functions of unknown genes by random matrix theory
Source: BMC Bioinformatics. 2007 Aug 14;8:299. doi: 10.1186/1471-2105-8-299 (PMC2212665; doi:10.1186/1471-2105-8-299)
Supplement: Additional file 2 — Notes for the understanding of random matrix theory and the description of modular networks obtained from microarray profiles of yeast, S. oneidensis and E. Coli. [file 1471-2105-8-299-S2.pdf]

SUPPLEMENTAL TABLES

Table S1: Functional predictions of unknown genes from yeast cell cycling dataset.

Systematic name	Syn	Predicted biological pathway/localization	Baikai's modules (2, 4)	Other predictions	SGD information	# of Links
YBL051C	PIN4	cytoplasmic, protein fate (folding, modification, destination)			cytoplasmic; mdt1, modulate DNA damage tolerance and G2/M cell cycle	2
YBR108W		cytoplasmic, protein fate (folding, modification, destination)	not present		synthetic sick with gim3,5, components of bovine prefoldin for tubulin folding and cytokinesis	3
YBR124W		cytoplasmic, protein fate (folding, modification, destination)	not present			2
YDR134C		amino acid biosynthesis or energy transport	not present			1
YOR248W		cytoplasmic, protein fate (folding, modification, destination)	cell cycle; module 63			2
YPL067C		cytoplasmic, protein fate (folding, modification, destination)	module 59			1
YKL153W		gluconeogenesis	module 60: glycolysis			1
YCR013C		gluconeogenesis	module 60: glycolysis			5
YDR279W	RNH202	cell cycle or cell fate	cell cycle; module 13		ribonuclease H2 subunit for DNA replication	2
YGR086C	PIL1	cell cycle	module 24	cell cycle (3);		1
YGR151C		cell cycle	cell cycle; module 15		synthetic lethal to Cde42p	12
YGR189C	CRH1	cell cycle	cell cycle; module 63		Putative glycosidase of the cell wall, may have a role in cell wall architecture	5
YGR221C	TOS2	cell cycle	cell cycle; module 63		Protein of unknown function that localizes to the bud neck and bud tip; potentially phosphorylated by Cdc28p	12
YBR070C		cell cycle	cell cycle; module 77			3
YBR071W		cell cycle	cell cycle; module 77	cell cycle (3);		1
YHR098C	SFB3	metabolism			Member of the Sec24p family; forms a complex, with Sec23p, that is involved in sorting of Pma1p into COPII vesicles; peripheral ER membrane protein; potential Cdc28p substrate	1
YHR149C		cell cycle	cell cycle; module 77		Protein of unknown function found in the bud tip and bud	1

					neck, potential Cdc28p substrate	
YBR089W		cell cycle	cell cycle; module 77			9
YIL158W		cell cycle	module 40			6
YJL051W		cell cycle	module 63		localized to the bud tip	6
YJL181W		cell cycle	cell cycle; module 77			3
YKR012C		cell cycle	mating; module 77			3
YKR013W	PRY2	cell cycle	cell cycle; module 82			9
YKR077W		cell cycle	cell cycle; module 82			2
YKR090W	PXL1	cell cycle	cell cycle; module 82		required for selection and/or maintenance of polarized growth sites, may modulate signaling by the GTPases Cdc42p and Rho1p; has similarity to metazoan paxillin	1
YLL022C	HIF1	cell cycle	cell cycle; module 82		Non-essential component of the HAT-B histone acetyltransferase complex (Hat1p-Hat2p-Hif1p), localized to the nucleus; has a role in telomeric silencing	14
YLR049C		cell cycle	cell cycle; module 85			3
YLR154C	RNH203	cell cycle	cell cycle		Ribonuclease H2 subunit, required for RNase H2 activity	1
YLR183C	TOS4	cell cycle	cell cycle; module 36		Transcription factor; promoters of some genes involved in pheromone response and cell cycle;	25
YLR190W	MMR1	cytokinesis	module 63: cell wall integrity		localized to small buds, bud neck, and incipient bud sites; mRNA is targeted to the bud via the mRNA transport system involving She2p	11
YML119W		cell cycle	module 47		potential Cdc28p substrate	10
YMR031C		metabolism	not present			1
YCL012W		cytokinesis or cell cycle	module 64			1
YCL013W		cytokinesis or cell cycle	module 64		"=YCL012W"	1
YNL058C		cell cycle	module 42	Cell cycle (1)	potential Cdc28p substrate	6
YCL022C		cell cycle	cell cycle; module 13			5
YOL007C		cell cycle	cell cycle; module 82		structural component of the chitin synthase 3 complex	19
YOL017W	ESC8	cell cycle	cell cycle; module 77		Protein involved in telomeric and mating-type locus silencing, interacts with Sir2p and also interacts with the Gal11p, which is a component	19

					of the RNA pol II mediator complex	
YOL019W		cell cycle	cell cycle; module 82			4
YCL060C		cell cycle	cell cycle; module 13; 15; 77		S-phase checkpoint protein found at replication forks, required for DNA replication; also required for Rad53p activation during DNA replication stress, where it forms a replication-pausing complex with Tof1p and is phosphorylated by Mec1p	10
YOR066W		cell cycle	module 42		potential Cdc28p substrate	2
YOR112W		metabolism	not present			1
YPL208W		cell cycle	cell cycle; module 77			7
YPR174C		cell cycle	cell cycle; module 82		potential Cdc28p substrate	15
YDL157C		cell cycle	cell cycle			1
YLL064C		stress response	not present			2
YKL177W		cell differentiation	not present			1
YPL158C		cytokinesis or cell fate	module 42			2
YDL117W	CYK3	cytokinesis	module 42		SH3-domain protein located in the mother-bud neck and the cytokinetic actin ring; mutant phenotype and genetic interactions suggest a role in cytokinesis	1
YOR135C		energy transport	module 21		exhibits growth defect on a non-fermentable (respiratory) carbon source	3
YDR361C	BCP1	ribosome biogenesis	protein synthesis			9
YDR413C		ribosome biogenesis	not present			3
YDR417C		protein synthesis	protein synthesis			5
YDR494W	RSM28	mitochondrial protein	module 12		Mitochondrial ribosomal protein of the small subunit	5
YGL102C		protein synthesis	protein synthesis			5
YGR123C	PPT1	ribosome biogenesis	protein synthesis		Protein serine/threonine phosphatase with similarity to human phosphatase PP5	8
YGR173W	GIR1	ribosome biogenesis	protein synthesis		GTPase, interacts with ribosomes	5
YGR187C	HGH1	ribosome biogenesis	protein synthesis			2
YBR056W		metabolism	stress			5
YGR248W	SOL4	metabolism	stress; module 34		6-phosphogluconolactonase activity by sequence analysis	1
YHL021C	FMP12	stress response	stress; module 34			8
YHR052W	CIC1	ribosome biogenesis	protein			2

			synthesis; module 10			
YHR116W		mitochondrial protein	module 12		Protein that functions in mitochondrial copper homeostasis and is essential for functional cytochrome oxidase expression; homologous to COX17, localized to the mitochondrial intermembrane space	1
YIL127C		ribosome biogenesis	protein synthesis			26
YJL009W		ribosome biogenesis	not present			1
YJL122W		ribosome biogenesis	protein synthesis			23
YKL030W		mitochondrial protein	not present			1
YKL169C		mitochondrial protein	module 12			1
YAL036C	FUN11	ribosome biogenesis	protein synthesis		RiBosome interacting Gtpase	6
YKR016W	FMP13	respiration	module 5: energy transport			
YKR047W		protein synthesis	not present		synthetic lethal to Bni1p involved in spindle formation	1
YLL037W		metabolism	not present			2
YLR016C		ribosome biogenesis	not present			1
YLR051C		ribosome biogenesis	protein synthesis			3
YLR062C	BUD28	protein synthesis	protein synthesis			2
YLR074C	BUD20	ribosome biogenesis	protein synthesis		Protein involved in bud-site selection; diploid mutants display a random budding pattern instead of the wild-type bipolar pattern	11
YLR090W	XDJ1	mitochondrial protein	not present		mitochondrial protein	2
YLR149C		metabolism	stress; module 37			1
YLR196W	PWP1	ribosome biogenesis	protein synthesis; module 10		nucleolar protein	16
YLR198C		ribosome biogenesis	protein synthesis			2
YLR217W		protein chaperone, response to heat shock	module 50: protein chaperone			1
YLR221C	RSA3	ribosome biogenesis	protein synthesis		Protein with a likely role in ribosomal maturation, required for accumulation of wild-type levels of large (60S) ribosomal subunits; binds to the helicase Dbp6p in pre-60S ribosomal particles in the nucleolus	13
YLR257W		mitochondrial protein	not present			1

YLR270W	DCS1	metabolism	stress		interact with stress response protein Nth1p	1
YLR339C		protein synthesis	module 70			2
YLR397C	AFG2	ribosome biogenesis	module 71		ATPase of the CDC48/PAS1/SEC18 (AAA) family, forms a hexameric complex; may be involved in degradation of aberrant mRNAs	4
YLR449W	FPR4	ribosome biogenesis	protein synthesis		Nuclear protein, putative peptidyl-prolyl cis-trans isomerase (PPIase) with similarity to Fpr3p; overproduction suppresses the growth defect resulting from the absence of E3 ubiquitin-protein ligase Tom1p	18
YBR266C		ribosome biogenesis	protein synthesis		endocytosis	4
YBR271W		ribosome biogenesis	protein synthesis			4
YML128C	MSC1	metabolism	stress; module 34		mutant has sporulation defect	1
YMR014W	BUD22	ribosome biogenesis	protein synthesis		Protein involved in bud-site selection	19
YMR269W		ribosome biogenesis	not present		protein possibly involved in protein synthesis	6
YMR290C	HAS1	ribosome biogenesis	protein synthesis; module 10		Putative ATP-dependent RNA helicase; localizes to both the nuclear periphery and nucleolus; highly enriched in nuclear pore complex fractions	17
YNL023C	FAP1	cell cycle	not present		Protein that binds to Fpr1p (FKBP12), conferring rapamycin resistance by competing with rapamycin for Fpr1p binding; has similarity to putative transcription factors, including D. melanogaster shuttle craft and human NFX1	2
YNL050C		ribosome biogenesis	not present			8
YNL132W	KRE33	ribosome biogenesis	protein synthesis; module 10			12
YNL174W		ribosome biogenesis	protein synthesis; module 10			1
YNR046W		ribosome biogenesis	protein synthesis		nucleolar protein	8
YNR054C		ribosome biogenesis	protein synthesis		transcription regulator activity	1
YCL042W		metabolism	stress			1
YOL022C		ribosome biogenesis	protein synthesis			8
YOL032W		mitochondrial protein	stress;			1

			module 56			
YOL048C		cytoplasmic, protein or nucleotide degradation	stress			2
YOL079W		ribosome biogenesis	not present			6
YOL124C		ribosome biogenesis	protein synthesis			6
YOR042W	CUE5	mitochondrial protein	module 35		Protein containing a CUE domain that binds ubiquitin, which may facilitate intramolecular monoubiquitination	1
YOR051C		ribosome biogenesis	not present			1
YOR090C	PTC5	mitochondrial protein	not present		Phosphatase type Two C	1
YOR146W		ribosome biogenesis	not present			16
YOR154W		ribosome biogenesis	not present			7
YOR169C		ribosome biogenesis	not present			2
YOR277C		ribosome biogenesis	module 41			2
YPL044C		ribosome biogenesis	protein synthesis; module 76			6
YCR016W		ribosome biogenesis	protein synthesis		nucleolar protein	10
YPL142C		protein synthesis	protein synthesis			2
YPL146C		ribosome biogenesis	protein synthesis		nucleolar protein	12
YPL202C	AFT2	ribosome biogenesis	not present		Activator of Iron (Fe) Transcription; response to oxidative stress	2
YPL207W		ribosome biogenesis	protein synthesis			8
YPL212C	PUS1	ribosome biogenesis	protein synthesis		Involved in tRNA biogenesis	1
YPL225W		proteolytic degradation	not present			1
YPL238C		ribosome biogenesis	protein synthesis			1
YPL239W	YAR1	ribosome biogenesis	protein synthesis		Null mutant is viable, grow slowly at low temperature	5
YPL273W	SAM4	metabolism	amino acid metabolism; module 68		sulfur amino acid metabolism	1
YPR048W	TAH18	ribosome biogenesis	not present			2
YPR136C		ribosome biogenesis	module 76			1
YPR142C		ribosome biogenesis	not present			1
YPR143W		ribosome biogenesis	protein synthesis		nucleolar protein	7
YPR169W	JIP5	ribosome biogenesis	not present		nucleolar protein; interact with ubiquitin-ligase Bre1p	7
YCR051W		ribosome biogenesis	module 70			1
YCR055C		ribosome biogenesis	protein synthesis		"=PWP2"	9
YCR072C		ribosome biogenesis	protein synthesis		co-purified with 60S ribosome subunit	9

YCR096C	HMRA2	transcriptional regulator, phenomone response	not present		Silenced copy of A2, encoding a protein of with similarity to Alpha2p that is required along with A1p for inhibiting expression of the HO endonuclease in a/alpha HO/HO diploid cells with an active mating-type interconversion system	2
YDL023C	SRF4	metabolism	not present		unlike to encode a protein	1
YDL063C		ribosome biogenesis	ribosome biogenesis			17
YDR056C		metabolism	protein synthesis		synthetic lethal to Nbp2p, a Nap1p-binding protein	1
YDR152W	GIR2	ribosome biogenesis	not present		Genetically Interacts with Ribosomal genes	4

Table S2: Functional predictions of unknown genes from *Shewanella* heat/cold shock dataset.

Gene designation	Prediction	TIGR Annotation	# of Links
SO1789	energy metabolism; transport	conserved hypothetical protein	1
SO2391	energy metabolism; transport	conserved hypothetical protein	1
SO3725	central intermediate metabolism; protein modification	hypothetical protein	3
SO4150	sulfur metabolism	transporter, putative	2
SO0119	metabolism	conserved hypothetical protein	2
SO0126	metabolism	conserved hypothetical protein	3
SO2011	metabolism	conserved hypothetical protein	3
SO2017	heat shcok response	conserved hypothetical protein	8
SO2042	heat shcok response	conserved hypothetical protein	3
SO2064	flagellar biosynthesis	conserved domain protein	3
SO2354	metabolism	conserved hypothetical protein	3
SO2375	metabolism	membrane protein, putative	8
SO2474	metabolism	carbonic anhydrase family protein	3
SO2500	flagellar biosynthesis	conserved hypothetical protein	1
SO3000	flagellar biosynthesis	conserved hypothetical protein	5
SO3015	flagellar biosynthesis	conserved hypothetical protein	3
SO3164	metabolism	conserved hypothetical protein	3
SO3288	flagellar biosynthesis	Transglycosylase, Slt family	2
SO3298	metabolism	conserved hypothetical protein	10
SO3336	metabolism	hypothetical protein	4
SO3896	heat shcok response	outer membrane porin, putative	3
SO4492	metabolism	conserved hypothetical protein	2
SO4556	metabolism	transcriptional regulator, LysR family	2
SO4565	flagellar biosynthesis	transporter, putative	3
SO0490	metabolism	transcriptional regulator	3
SO0591	metabolism	conserved hypothetical protein	3
SO0595	metabolism	hypothetical protein	2
DsbD	metabolism	thiol:disulfide interchange protein	5
SO1125	heat shcok response	Integral membrane domain protein	3
SO2862	amino acid metabolism	HDIG domain protein	2
SO4051	amino acid metabolism	HlyD family secretion protein	2
SO3696	sulfur metabolism	hypothetical protein	1

Table S3: Functional predictions of unknown genes from *E.coli* datasets.

Gene designation	Prediction	Annotation*	# of Links
b1155	biosynthesis of small molecules	hypothetical protein	2
b1156	biosynthesis of small molecules	hypothetical protein	1
yafJ	biosynthesis of small molecules	conserved protein with N-terminal nucleophile aminohydrolase domain	1
b0224	biosynthesis of small molecules	hypothetical protein	2
b2353	biosynthesis of small molecules	hypothetical protein	2
b2354	biosynthesis of small molecules	hypothetical protein	2
b2596	degradation of amino acids	hypothetical protein	2
b2853	degradation of amino acids	hypothetical protein	2
b3263	degradation of amino acids	hypothetical protein	2
cspH	degradation of small molecules	cold shock-like protein, nucleic acid-binding domain	2
b1088	protein synthesis	conserved protein of unknown function	5
b1815	transport of small molecule	conserved protein of unknown function	2
b1933	function related to cell surface structure	hypothetical protein	5
yaeC	metabolism, cell surface structure	D-methionine transport protein (ABC superfamily, peri_bind)	8
b1983	nucleotide metabolism	hypothetical protein	2
yejM	translation, post-translational modification	putative sulfatase	12
b0303	carbon compound catabolism	hypothetical protein	2
agaA	post-translational modification	putative N-acetylgalactosamine-6-phosphate deacetylase	13
envR	function related to cell surface structure	transcriptional repressor for multiple drug efflux pump (TetR/AcrR family)	3
rhlB	amino acid metabolism	putative ATP-dependent helicase with nucleoside triP hydrolase domain	3
yihK	nucleotide metabolism	putative GTP-binding factor	4
yajC	amino acid biosynthesis	preprotein translocase, auxillary membrane component (General Secretory Pathway)	6
b4066	function related to cell surface structure	hypothetical protein	2
b4103	function related to cell surface structure	hypothetical protein	4
b4243	protein translation and modification	hypothetical protein	5
ybgF	protein synthesis	putative periplasmic protein with protein prenylyltransferase domain	9
b1194	flagellar biosynthesis	hypothetical protein	2
b1148	carbon compound catabolism	hypothetical protein	3
b1505	transport protein	putative outer membrane protein	9
b1644	transport protein	putative multidrug resistance membrane protein	3
b1645	transport protein	hypothetical protein	2

b1673	transport of small molecule	putative aldehyde ferridoxin oxidoreductase	7
yeeP	transport protein	CP4-44 prophage; putative GTP-binding factor	2
b2459	energy metabolism; transport protein	putative cobalamin adenosyltransferase with winged helix domain, in ethanolamine utilization	7
b2506	function related to cell surface structure	putative membrane protein	3
yqcB	energy metabolism	pseudouridine synthase	6
yggR	energy metabolism; transport protein	putative protein transport	3
yghD	transport protein	putative secretion pathway protein	5
b2970	transport protein	putative protein exporter (General Secretory Pathway)	4
b2973	energy metabolism; transport protein	hypothetical protein	5
yraL	energy metabolism	putative enzyme with Cobalt precorrin-4 methyltransferase domain	2
yhfP	transport protein	conserved protein with Xylose isomerase-like domain	6
yhjQ	transport protein; amin acid metabolism	putative cell division protein, nucleotide triphosphate hydrolase domain	4
b3832	transport protein	hypothetical protein	8
b4212	energy metabolism; transport protein	hypothetical protein	6
b4256	transport protein	hypothetical protein	3
b0960	transport protein	putative efflux transport protein (PET family)	2

*: annotation is based on information from TIGR's *E.coli* annotation database and personal communication with Margrethe Serres, Marine Biological Laboratory, Woods Hole, MA, 02543)

References

1. **Bar-Joseph, Z., G. K. Gerber, T. I. Lee, N. J. Rinaldi, J. Y. Yoo, F. Robert, D. B. Gordon, E. Fraenkel, T. S. Jaakkola, R. A. Young, and D. K. Gifford.** 2003. Computational discovery of gene modules and regulatory networks. Nat Biotechnol **21:**1337-42.
2. **Bergmann, S., J. Ihmels, and N. Barkai.** 2003. Iterative signature algorithm for the analysis of large-scale gene expression data. Phys Rev E Stat Nonlin Soft Matter Phys **67:**031902.
3. **Fellenberg, K., N. C. Hauser, B. Brors, A. Neutzner, J. D. Hoheisel, and M. Vingron.** 2001. Correspondence analysis applied to microarray data. Proc Natl Acad Sci U S A **98:**10781-6.
4. **Ihmels, J., G. Friedlander, S. Bergmann, O. Sarig, Y. Ziv, and N. Barkai.** 2002. Revealing modular organization in the yeast transcriptional network. Nat Genet **31:**370-7.
