# Supplementary material for: Constructing gene co-expression networks and predicting functions of unknown genes by random matrix theory
Source: BMC Bioinformatics. 2007 Aug 14;8:299. doi: 10.1186/1471-2105-8-299 (PMC2212665; doi:10.1186/1471-2105-8-299)
Supplement: Additional file 3 — Tables showed the 215 functionally unknown genes from yeast, E. coli and S. oneidensis are predicted by the gene co-expression networks using guilt-by-association principle. [file 1471-2105-8-299-S3.pdf]

Supplementary Notes (A-D)

A. Introduction to random matrix theory (RMT)

RMT, initially proposed by Wigner for studying the spectrum of complex nuclei (10), is very powerful in identifying and modeling phase transitions associated with disorder and noise in statistical physics and materials science. It has been successfully applied to investigate a wide variety of complex systems such as large atoms (4, 9), metal insulator transitions in disorder systems (1, 6), quasiperiodic systems (11, 12), and chaotic systems (3, 5). One of the essential elements in the random matrix theory is that eigenvalue fluctuations of real random matrices follow two different universal laws depending on the correlation property of eigenvalues. Strong correlation of eigenvalues leads to eigenvalue fluctuations described by Gaussian orthogonal ensemble (GOE) statistics. On the other hand, eigenvalue fluctuations follow Poisson statistics if there is no correlation between eigenvalues. A typical example of the GOE level fluctuation is a random matrix with a random distribution of all matrix elements, where the non-zero off-diagonal elements represent mutual interactions of the diagonal elements that induce strong correlations of eigenvalues. In contrast, level fluctuation of a matrix with non-zero values only for its block-diagonal parts would follow Poisson statistics, because the absence of interaction between these block-diagonal parts results in zero or less pronounced correlations between engenvalues of the matrix.

In general, the density of levels (or eigenvalues) of a matrix varies with its level $E_i\,(i = 1,2,3,\ldots N)$, where *N* is the order of the matrix. In order to observe the universal level fluctuations of different matrices, RMT requires spectral unfolding to have constant density of levels. To fulfill this, one can replace levels E_i by $e_i = N_{av}(\lambda_i)$, where $N_{av}(\lambda_i)$ is the smoothed integrated density of levels that can be obtained by fitting the original integrated density of levels to a cubic spline or by local density average. With the unfolded levels, one can calculate the level spacing distribution $P(s)$, which is defined as the probability density of level spacing $s = e_{i+1} - e_i$. For Poisson statistics, one has $P_P(s) = \exp(-s)$. For the GOE statistics, $P(s)$ closely follows the Wigner-Dyson distribution $P_{GOE} \approx \frac{1}{2}\pi s \exp(-\pi s^2/4)$.

B. Summary of modules revealed in yeast cell cycling data

The following is a brief description of the modules observed in Fig. 2. The yeast cell cycling dataset contains expression profiles for about 6,200 yeast genes in six cell cycle related experiments and a total of 77 time points. We only included genes for which at least 70 time points out of 77 were available, resulting in 5,293 genes for this study.

Module 1: All of the 3 known proteins are annotated in protein fate, though the biological functions are different: *APL3* in vesicle-mediated transport, *ALG1* in N-linked glycosylation, and *ADY3* in protein complex assembly.
Module 2: There are four genes in this module. *LEU9* and *HOM2* are involved in amino acid biosynthesis. *ATP1* is an ATP synthase. The processes of energy transport and amino acid metabolism are often seen to be coordinated (other examples are module 5 and 17). It is conceivable that amino acid metabolism requires many intermediates of the TCA cycle.
Module 3: All five proteins participate in galactose metabolism. Notably, all five proteins can be regulated by the transcriptional regulator Gal4p (7).
Module 4: There are 16 genes in this module. Seven have annotated functions in protein fate, specifically, in vesicle transport or glycosylation. Five could be annotated in cell fate, specifically, cell wall organization. These could be two closely related biological processes since many proteins in cell wall organization need to be glycosylated and transported. The five genes of cell wall organization

associate with each other intimately. Indeed, they segregate from others at a cutoff of 0.82 to form an independent module.

Module 5: Out of the 6 known proteins in module 5, four are predicted to be involved in amino acid or lipid transport and two in the TCA cycle.

Module 6: It has five proteins. Except one unknown gene, they have different functions, but all are involved in mating. *SST2* is involved in signal transduction, *KAR4* is a transcriptional factor, and *FUS1* and *AGA1* are required for conjugation with cellular fusion.

Module 7: All five known genes have annotated functions in gluconeogenesis.

Module 8: All three genes in this module are of unknown function Thus, no analysis was made.

Module 9: Module 9 is a big module comprised of 187 genes. It can be visually divided into three sub-modules: Y'-cluster, cell cycle regulator and histones. Y'-cluster is separated from cell cycle regulators at the cutoff 0.81. Y'-cluster is composed of many unknown genes and some genes involved in telomerase-independent telomere maintenance. All genes in Y'-cluster are located near the end of chromosomes. It is unclear if all or just a few genes in Y'-cluster are co-regulated, because their sequence similarity is high. Although the functional linkage of Y'-cluster genes and cell cycle is not clearly established, there is a recent paper (8) reporting that defective telomeres have aberrant chromosome separation and segregation during mitosis, likely due to a checkpoint response.

For the 96 known genes in the cell cycle regulator sub-module, 63 are cell cycling regulators of mitosis (M), M→ G1 and G1 phases. For example, *MCM3*, *CDC47* and *CDC46* are part of the pre-replicative complex, an assembly of proteins formed between late mitosis and the G1/S phase. Also, additional 14 genes have annotated functions in cell fate determination, primarily budding or mating, which is consistent with knowledge that budding or mating is determined during G1 phase.

The third sub-module consists of 10 proteins including all 8 histone proteins and *HHO1*, a regulator of transcription and linker to other histones.

Module 9 disintegrated into two modules at a cutoff of 0.79. One is comprised of the Y'-cluster genes and cell cycle regulators, whereas the other one is comprised of histone proteins. Furthermore, module 11 disintegrated into another two modules at 0.81, which are exactly module of Y'-cluster genes and module of cell cycle regulators.

Module 10: There are only three genes in module 12. Two are members of the seripauperin protein/gene family and have been annotated in cell defense. One is unknown.

Module 11: Module 13 has four proteins. One is unknown and three are annotated in fungal cell differentiation.

Module 12: All four proteins in this module are clearly involved in protein synthesis. Specifically, three are ribosome proteins and one is translation elongation factor.

Module 13: It has 18 proteins, in with 9 are annotated in cell wall organization and cytoskeleton. Five are annotated in cell cycle and are involved in exit from mitosis and cell division, thus they are closely related to cell wall organization as required for cell division. For example, *ASH*1 shows strong association with *PIR1*, *EGT2* and *NIS1*, consistent with the observation that it is distributed to the bud tip at the anaphase in a cytoskeleton-dependent manner (2).

Module 14: Seven out of the 9 genes in this module participate in energy metabolism including TCA cycle, while one gene is annotated in amino acid metabolism.

Module 15: This module, which is the largest, contains 525 genes. Like module 9, it can be divided into several large functional sub-modules: ribosomal biogenesis, ribosomal proteins, mitochondrion and protein degradation. Their functional linkages are quite obvious because defective ribosomal biogenesis often leads to rapid depletion of ribosomal proteins. It is linked to mitochondria also, possibly due to the fact that there is an independent set of ribosomal proteins in mitochondria. Lastly, there is evidence demonstrating that cells repress ribosomal biosynthesis and activate protein degradation upon stress conditions (ref).

The ribosomal biogenesis sub-module has more than 200 proteins, most of which have been demonstrated experimentally in ribosomal biogenesis. Since ribosomal biogenesis is a very essential and complicated biological process, it is not unexpected to identify many other genes in such closely related processes as pre-mRNA splicing, Pol III transcription and translational initiation. Indeed, it has been well established that rRNA modification is carried out by snoRNAs, which is produced by either

pre-mRNA splicing or Pol III transcription. In addition, 5S rRNA is also synthesized by Pol III transcription.

In the sub-module of genes encoding ribosome proteins, 85% genes encode ribosomal proteins, in addition to several ribosome-associated proteins which are involved in translation.

The sub-module of mitochondria also contains 14 genes annotated in protein degradation. They might form another distinct functional sub-module.

At a cutoff of 0.79, module 15 disintegrates into two large modules and many smaller modules. These two large modules correspond to the sub-modules of ribosomal proteins and ribosomal biogenesis.

Notably, module 15 contains many unknown genes that are linked to the genes involved in ribosomal biogenesis via multiple links, thus their function can be predicted with high confidence (see Table S1 for details).

C. Summary of modules from *Shewanella oneidensis* heat/cold shock data

The RMT method was applied to *Shewanella oneidensis* whole-genome microarray dataset of 15 temperature stress response conditions: cells were shifted from 30^0C to 42^0C for 5, 15 and 25 minutes or to 8^0C for 5, 10, 20, 40, 80 and 160 minutes, or to 15^0C for 5, 10, 20, 40, 80 and 160 minutes. Threshold was determined to be 0.95.

Module 1: This module of six genes is dedicated to energy transport and TCA cycle.
Module 2: All 4 genes are involved in energy metabolism.
Module 3: Two out of four proteins are transport proteins, while the other two apparently participate in energy production and might thus facilitate the transport.
Module 4: Most of the genes in this compact module are involved in the ulfite transport and metabolism.
Module 5: It is the largest module of 401 genes with apparently diverse functional categories. The major functional categories in the module are genes involved in energy transport and amino acid transport/biosynthesis. Two other major functional categories are heat shock response and chemotaxis, as represented in yellow and tan, respectively. Most genes in the chemotaxis category are flagellar components.
Module 6: Out of six known genes, four are involved in biosynthesis of small molecules.
Module 7: All of the 3 known genes are involved in sulfur metabolism.

D. Summary of modules from *E.coli* datasets

A total of 99 experimental datasets were downloaded from the University of Oklahoma *E. coli* gene expression database. RMT-based method determines a threshold of 0.855 for modular analysis.

Module 1: There are 3 known genes and 1 unknown gene in this module. *fdnG* and *agaB* are involved in carbohydrate metabolism, whereas *htgA* is a putative regulator of sigma 32 heat shock-regulated promoters.
Module 2: All 6 genes are components of a putative transport system.
Module 3: Most genes in this 12-gene module are unknown.
Module 4: All three genes are putative regulators.
Module 5: All 9 genes encode IS5 transposases.
Module 6: Six out of 9 genes are unknown. Two known genes (*dsdC* and *tdcR*) are regulators for the degradation of amino acids, whereas the other known gene (*fimE*, #377) is a regulator involved in phase variation.
Module 7: All 3 genes in Module 9 are putative transposases.
Module 8: All 10 genes are involved in two closely related processes, energy transport and amino acid transport/biosynthesis.

Module 9: Three of 4 genes are involved in energy transport; the last one is an outer membrane protein induced by carbon starvation.
Module 10: Out of a total of 6 genes, 4 genes are annotated in diversified processes: one in DNA repair, one in surface structure, one in aerobic respiration sensor and the fourth in transport.
Module 11 and 12: Almost all the genes clustering in these modules are of unknown function; thus, further analysis was not conducted.
Module 13: All genes are unknown.
Module 14: It is a big module of 345 genes. It is mainly composed of ribosome genes and ribosome-associated proteins including many aminoacyl tRNA synthases, as presented in red. Its related processes include genes involved in amino acid synthesis and mRNA synthesis. Besides these two categories, there are many genes involved in metabolism, including genes in nucleotide metabolism and energy transport. This close association of protein synthesis and metabolism is also seen in the yeast cell cycle dataset.
Module 15: Almost all genes are unknown, and no further anlysis was done.
Module 16: All 19 genes are components of flagellar assembly.
Module 17: All 3 genes are involved in phospholipid metabolism.
Module 18: This is mainly composed of putative regulatory genes. Thirty out of 42 known genes are putative regulatory genes. It is notable that 28 of them form a compact cluster.
Module 19: The function of these 3 genes is diverse. One is unknown, one in anaerobic energy metabolism, and the other one in heat shock response.
Module 20: Two out of 3 genes are responsible for cell surface structure.
Module 21 and 22: Almost all genes are unknown; thus, it was not further analyzed.
Module 23: In this 4-gene module, three are involved in sensing environmental changes and regulate DNA replication and repairing.
Module 24: All genes are unknown.
Module 25: This module contains 285 genes. The largest functional category is transport of small molecules and energy metabolism. There are a lot of genes involved in transporting sugar to the cell surface. Meanwhile, there are some genes responsive to stress condition (presented in yellow), A likely explanation is that transporting sugar to the cell surface could help to resist external stress.
Module 26: All of 3 genes are unknown.
Module 27: All 5 genes seem to be involved in the transport of maltose and galactose. Indeed, all genes, except *mglB*, are clustered in one operon, thus coexpression is observed.
Module 28: All 6 genes are putative transport genes.
Module 29: Almost all known genes except one (*surE*) are annotated in either energy metabolism or transport of nucleotide.
Module 30: All 3 genes are involved in transport of dipeptides and organized in the same operon.

References:

1. **Alt'shuler, B. L., and B. I. Shklovskii.** 1986. Repulsion of energy-levels and the conductance of small metallic samples. Sov. Phys. JETP **64:**127.
2. **Bertrand, E., P. Chartrand, M. Schaefer, S. M. Shenoy, R. H. Singer, and R. M. Long.** 1998. Localization of ASH1 mRNA particles in living yeast. Mol Cell **2:**437-45.
3. **Bohigas, O., M. J. Giannoni, and C. Schmit.** 1984. Spectral properties of the laplactian and random matrix theories. Physical Review Letter **52:**1.
4. **Brody, T. A., J. Flores, J. B. French, P. A. Mello, A. Pandey, and S. S. M. Wong.** 1981. Random-matrix physics: spectrum and strength fluctuations. Rev. Mod. Phys **53:**385-480.
5. **Casati, G.** 1985. Chaotic Behavior in Quantum Systems: Theory and Application. Plenum Press, New York.

6. **Hofstetter, E., and M. Schreiber.** 1993. Statistical properties of the eigenvalue spectrum of the three-dimensional Anderson Hamiltonian. Physical Review. B. Condensed Matter. **48:**16979-16985.
7. **Ihmels, J., R. Levy, and N. Barkai.** 2004. Principles of transcriptional control in the metabolic network of Saccharomyces cerevisiae. Nat Biotechnol **22:**86-92.
8. **Lin, J., D. L. Smith, and E. H. Blackburn.** 2004. Mutant telomere sequences lead to impaired chromosome separation and a unique checkpoint response. Mol Biol Cell **15:**1623-34.
9. **Mehta, M. L.** 1967. Random Matrices and the statistical theory of energy levels. Academic Press.
10. **Wigner, E. P.** 1967. Random Matrices in Physics. SIAM Review **9:**1-23.
11. **Zhong, J. X., and T. Geisel.** 1999. Level fluctuations in quantum systems with multifractal eigenstates. Physical Review **E 59:**4071-4074.
12. **Zhong, J. X., U. Grimm, R. A. Romer, and M. Schreiber.** 1998. Level-spacing distribution of planar quasiperiodic tight-bindind models. Physical Review Letter **80:**3996-3999.
